# Cerebrospinal fluid and brain positron emission tomography measures of synaptic vesicle glycoprotein 2A: Biomarkers of synaptic density in Alzheimer's disease

**DOI:** 10.1002/alz.70344

**Published:** 2025-06-09

**Authors:** Adam P. Mecca, Nicholas J. Ashton, Ming‐Kai Chen, Ryan S. O'Dell, Takaya Toyonaga, Wenzhen Zhao, Juan J. Young, Elaheh Salardini, Kara A. Bates, Jocelyn Ra, Sam Goodcase, Jason A. Silva‐Rudberg, Nabeel B. Nabulsi, Ann Brinkmalm, Hlin Kvartsberg, Michael Schöll, Johanna Nilsson, Amy F. T. Arnsten, Yiyun Huang, Oskar Hansson, Henrik Zetterberg, Richard E. Carson, Kaj Blennow, Christopher H. van Dyck

**Affiliations:** ^1^ Alzheimer's Disease Research Unit, Yale School of Medicine New Haven Connecticut USA; ^2^ Department of Psychiatry Yale School of Medicine New Haven Connecticut USA; ^3^ Department of Psychiatry and Neurochemistry Institute of Neuroscience and Physiology, The Sahlgrenska Academy at the University of Gothenburg Mölndal Sweden; ^4^ Department of Radiology and Biomedical Imaging Yale School of Medicine New Haven Connecticut USA; ^5^ Clinical Neurochemistry Lab Sahlgrenska University Hospital Mölndal Sweden; ^6^ Department of Neuroscience Yale School of Medicine New Haven Connecticut USA; ^7^ Clinical Memory Research Unit Department of Clinical Sciences Malmö Lund University Lund Sweden; ^8^ Paris Brain Institute, ICM, Pitié‐Salpêtrière Hospital, Sorbonne University Paris France; ^9^ Neurodegenerative Disorder Research Center Division of Life Sciences and Medicine and Department of Neurology Institute on Aging and Brain Disorders, University of Science and Technology of China and First Affiliated Hospital of USTC Hefei China; ^10^ Department of Neurology Yale School of Medicine New Haven Connecticut USA

**Keywords:** Alzheimer's disease, biomarkers, CSF, SV2A, synaptic density

## Abstract

**INTRODUCTION:**

Positron emission tomography (PET) imaging with ligands for synaptic vesicle glycoprotein 2A (SV2A) has emerged as a promising methodology for measuring synaptic density in Alzheimer's disease (AD). We investigated associations between SV2A concentrations in the brain and cerebrospinal fluid (CSF).

**METHODS:**

Twenty‐one participants with early AD and 7 cognitively normal (CN) individuals underwent [^11^C]UCB‐J PET. We used a novel enzyme‐linked immunosorbent assay (ELISA) to measure CSF SV2A. Other synaptic and axonal proteins were also measured in CSF.

**RESULTS:**

CSF SV2A was lower in AD compared to CN participants. Within the AD group, CSF SV2A was highly correlated with SV2A PET. By contrast, other CSF proteins were generally higher in participants with AD and not associated with SV2A PET.

**DISCUSSION:**

We report a novel CSF assay for SV2A that is strongly correlated with the PET measurement of SV2A. Our results suggest that CSF SV2A may serve as a biomarker for synaptic density in AD.

**Highlights:**

- Synaptic vesicle glycoprotein 2A (SV2A) measured by a novel cerebrospinal fluid (CSF) enzyme‐linked immunosorbent assay (ELISA) was lower in participants with symptomatic Alzheimer's disease (AD).
- CSF SV2A was highly correlated with SV2A measured by positron emission tomography (PET) in participants with AD.
- Other CSF synaptic/axonal proteins were not significantly associated with SV2A PET.
- CSF SV2A may serve as a biomarker for synaptic density in AD.

## 1 BACKGROUND

Development of biomarkers to assess disease progression and therapeutic efficacy is an important goal of ongoing Alzheimer's disease (AD) research. Because synaptic loss is a major pathological correlate of cognitive impairment in AD^1^, ^2^ and synaptic protein levels are generally decreased in cortical areas in AD brain tissue,^3^ synaptic and axonal proteins are potential candidates for fluid and imaging biomarker development.

Advances in positron emission tomography (PET) have allowed in vivo quantification of brain synaptic density using tracers for synaptic vesicle glycoprotein 2A (SV2A), a presynaptic protein expressed in nearly all synapses.^4^, ^5^ Consistent with pathological studies, several human imaging studies have shown that synaptic density is lower in participants with AD using the SV2A radioligands [^11^C]UCB‐J^6^, ^7^ and [^18^F]UCB‐H.^8^ Recently, we reported that in vivo synaptic density measured with SV2A PET is strongly correlated with cognitive performance in a sample of individuals with AD.^9^ Additional PET synaptic imaging studies have also provided evidence for a cross‐sectional^10^, ^11^ and longitudinal^12^ association between synaptic loss and fibrillary tau accumulation in AD.

Development of SV2A PET may provide valuable information about regional patterns of synaptic loss that can be combined with studies of cerebrospinal fluid (CSF) biomarkers of synaptic and axonal integrity to gain insight into the neurobiology of AD. CSF protein assays for presynaptic, postsynaptic, and axonal protein targets are promising biomarkers for AD. A number of these proteins, including neurofilament light chain (NFL), growth‐associated protein 43 (GAP‐43), neurogranin, synaptosomal‐associated protein 25 (SNAP25), and synaptotagmin‐1, are elevated in participants with AD.^13^, ^14^, ^15^, ^16^, ^17^ However, the relationship between brain synaptic density and fluid measures of synaptic or axonal integrity has yet to be determined. Measurement of fluid markers of synaptic and neuronal injury could provide complementary information in studies of AD neurobiology and widely accessible biomarkers of AD.

In this study, we first examined the relationship between PET and CSF measures of SV2A, a candidate marker of synaptic density. We aimed to test the hypothesis that a novel CSF measure of SV2A is significantly correlated with a global PET measure of SV2A binding in AD, a disease in which synaptic loss is a pathologic hallmark. We also examined the associations of SV2A PET with other CSF markers of synaptic and axonal integrity. Finally, we investigated the regional patterns of association between SV2A PET and CSF biomarkers to explore the relationship with each fluid biomarker and reginal synaptic loss.

## 2 METHODS

Detailed methods are available in the online Supplementary Material.

### 2.1 Study participants and design

SV2A PET Study Cohort: Participants aged 50–85 years were screened for eligibility as previously described.^18^ Individuals with dementia met diagnostic criteria for probable AD dementia,^19^ had a Clinical Dementia Rating (CDR) global score of 0.5–1.0, and a Mini‐Mental State Examination (MMSE) score of ≤ 26. Participants with mild cognitive impairment (MCI) met diagnostic criteria for amnestic MCI,^20^ had a CDR‐global score of 0.5, and a MMSE score of 24–30, inclusive. Participants with dementia and MCI were required to demonstrate impaired episodic memory, as evidenced by a logical memory (LM) II score 1.5 standard deviations (SDs) below an education‐adjusted norm. Cognitively normal (CN) participants were required to have a CDR‐global score of 0, a MMSE score of > 26, and a normal education‐adjusted LMII score. All participants received a PET scan with [^11^C]Pittsburgh Compound B ([^11^C]PiB) to evaluate the accumulation of brain amyloid β (Aβ), and a PET scan with [^11^C]UCB‐J to measure SV2A binding. Participants with dementia and MCI were required to be Aβ positive, and CN participants were required to be Aβ negative if both visual and quantitative criteria were met. Visual reading was performed by an experienced reader (A.P.M., M.K.C., or R.S.O.), and quantitative criteria required a [^11^C]PiB cerebral‐to‐cerebellar distribution volume ratio (*DVR*) of 1.40 or more in at least 1 AD‐affected region of interest (ROI).^21^, ^22^

**RESEARCH IN CONTEXT**

1. **Systematic review**: Positron emission tomography (PET) imaging with ligands for synaptic vesicle glycoprotein 2A (SV2A) has emerged as a promising methodology for measuring synaptic density in Alzheimer's disease (AD). This study compared the results of a novel cerebrospinal fluid (CSF) assay for SV2A with those of SV2A PET in the same participants.
2. **Interpretation**: SV2A measured in CSF was lower in participants with clinical symptoms of AD compared to cognitively healthy participants. Within the AD group, CSF SV2A was highly correlated with SV2A binding measured via PET. Other CSF synaptic and axonal proteins were not associated with SV2A PET. These results suggest that CSF SV2A may serve as a biomarker for synaptic density in AD.
3. **Future directions**: Larger and longitudinal studies are needed to confirm the relationship between CSF biomarkers of synaptic integrity and regional patterns of synaptic loss measured with SV2A PET.

CSF SV2A discovery cohorts: Participants from the CSF discovery cohort 1 and discovery cohort 2 were recruited from Sahlgrenska University Hospital and Memory Clinic at the University of Lund, respectively. Discovery cohort 1 included patients with AD (*n* = 20) based on clinical evaluation and AD CSF biomarker profiles (Aβ1‐42 < 530 ng/L, p‐tau181 > 60 ng/L, and t‐tau > 350 ng/L), and cognitively unimpaired control participants (CU, *n* = 20) with CSF biomarker levels in the normal ranges. Discovery cohort 2 included patients with AD (*n* = 31) and CU control participants (Aβ+ *n* = 14 and Aβ‐ *n* = 42). Participants with clinical AD were biomarker positive based on the International Working Group‐2 (IWG‐2) criterion^23^ which included a low concentration of CSF Aβ42 (< 550 ng/L) and a high level of t‐tau (> 400 ng/L) or p‐tau181 (> 80 ng/L). CU controls with Aβ42 < 550 ng/L were classified as CU Aβ+. All CU participants had tau biomarkers within normal ranges.

All participants provided written informed consent as approved by the institutional Human Investigation Committees.

### 2.2 Brain imaging

T1‐weighted magnetic resonance imaging (MRI) was performed to define ROI and to perform partial volume correction (PVC) using the Iterative Yang (IY) approach.^24^, ^25^ PET scans were performed on the High Resolution Research Tomograph (207 slices, resolution < 3 mm full width half max).^26^ List‐mode data were reconstructed using the MOLAR algorithm^27^ with event‐by‐event motion correction based on an optical detector (Vicra, NDI Systems, Waterloo, Canada).^28^ Dynamic [^11^C]PiB scans were acquired for 90 min following a bolus of up to 555 MBq of tracer^22^ and dynamic [^11^C]UCB‐J scans were acquired for 60 or 90 min after administration of a bolus of up to 740 MBq.^29^ Software motion correction was applied to the dynamic PET images using a mutual‐information algorithm (FSL‐FLIRT) to perform frame‐by‐frame registration to a summed image (0–10 min). A summed motion corrected PET image was registered to each MRI. Cortical reconstruction and volumetric segmentation was performed using FreeSurfer [version 6.0].^30^ Regions defined by the FreeSurfer segmentation were used for both PET and MRI analyses in native participant space. Brain volume was normalized using estimated total intracranial volume.^31^ A composite ROI of AD‐affected regions (prefrontal, lateral temporal, medial temporal, lateral parietal, anterior cingulate, posterior cingulate, precuneus, and lateral occipital) was defined (eTable 1).

### 2.3 Tracer kinetic modeling

For [^11^C]PiB image analysis, parametric images of *BP*_ND_ were generated using a simplified reference tissue model‐2 step (SRTM2)^32^ as previously described.^18^, ^22^ For [^11^C]UCB‐J image analysis, parametric images of *DVR* were generated using an SRTM2 from 0 to 60 min^32^ and whole cerebellum as the reference region.^7^, ^33^

### 2.4 CSF collection

Lumbar Puncture was performed under local anesthesia at interspace L3‐L4 using a 20 ga Quincke needle by gravity collection with the participant lying on their side. No tubing was used. Up to 30 mL of CSF was collected in 12 mL sterile culture tubes. Samples were centrifuged at 2200 rpm at room temperature for 15 min in an Eppendorf centrifuge in the original culture tubes and combined into one 50 mL polypropylene conical tube and gently inverted to mix. Then they were divided into 0.5 mL aliquots in 1 mL cryotube vials and stored at −80°C.

### 2.5 CSF protein assays

Aβ42, Aβ40, and tau phosphorylated at threonine‐181 (pTau181) in CSF were measured with a multiplex platform (xMAP; Luminex Corporation) with a fluorometric immunoassay (INNO‐BIA AlzBio3, Fujirebio Europe).^34^ NFL,^17^ GAP‐43,^16^ and neurogranin^15^ were measured in CSF using an in‐house enzyme‐linked immunosorbent assay (ELISA) method. SNAP25 (Total and Long),^14^ and synaptotagmin‐1^13^ were measured using affinity purification and mass spectrometry. SV2A in CSF was measured by an in‐house ELISA developed to target the N‐terminal region (aa2‐17). Detailed methods for this assay are reported in the online supplement.

### 2.6 CSF SV2A ELISA validation

Assay validation in discovery cohort 1 focused on intermediate precision, parallelism, and dilution linearity in three CSF pools determined to be high (2112 pg/mL), intermediate (1456 pg/mL), and low (750 pg/mL) in SV2A levels. The assay intermediate precision (14.5%) was determined by measuring the SV2A concentration in the three CSF pools, aliquoted and stored at −80°C, in 5–7 duplicates on three different occasions. For parallelism, CSF samples were analyzed undiluted or diluted (two‐fold and four‐fold), and the % recovery was calculated (eTable 2). Dilution linearity (eTable 3) was measured by spiking a known concentration of recombinant SV2A into the three CSF samples and quantifying undiluted or diluted (two‐fold and four‐fold). The lower limit of quantification (325 pg/mL) was calculated as the mean of 12 blank duplicates plus 10 times its SD. Discovery cohorts 1 and 2 were then used to assess for differences in CSF SV2A levels in Aβ+ participants with AD compared to CU (Aβ+ and Aβ‐) groups.

### 2.7 Statistical analyses

Statistical methods are detailed in the online supplement. Group comparisons were performed using χ^2^ tests for categorical variables and analysis of variance (ANOVA) or unpaired *t*‐tests for continuous variables. Univariate regression and Pearson's correlation were used to assess the association between CSF biomarkers and global or regional SV2A binding (*DVR*). Pearson's *r* (effect size) maps were created with the voxels in each region set uniformly to the calculated effect size. *p *< 0.05 was used as a threshold for significance.

## 3 RESULTS

### 3.1 CSF SV2A in discovery cohorts

Discovery cohorts 1 and 2 were assessed for group differences in CSF SV2A concentrations. In cohort 1, CSF SV2A was significantly lower in participants with AD (1072 ± 393 pg/mL, *n* = 20) compared to CU Aβ‐ participants (1679 ± 433 pg/mL, *n* = 20, unpaired *t*‐test, *p* < 0.0001, eFigure 1). In cohort 2, there was a significant difference in CSF SV2A among groups of participants with AD (1407 ± 538 pg/mL, *n* = 31), CU Aβ+ participants (2096 ± 382 pg/mL, *n* = 14), and CU Aβ‐ participants (2018 ± 410 pg/mL, *n* = 42, ANOVA, *p* < 0.0001, eFigure 2). Between‐group analyses indicated that CSF SV2A was significantly lower in the AD group, compared to either the CU Aβ+ group (unpaired *t*‐test, *p* < 0.0001) or the CU Aβ‐ group (unpaired *t*‐test, *p* < 0.0001). CSF SV2A was not different in the CU Aβ+ compared to the CU Aβ+ participants (unpaired *t*‐test, *p* = 0.54).

### 3.2 SV2A PET cohort participant characteristics

The study sample with both CSF and PET SV2A measures included 28 participants—21 with AD (17 with mild dementia, 4 with MCI), and 7 who were CN—whose characteristics are shown in Table 1. The sample is a subset of participants included in previous analyses that did not investigate CSF biomarkers.^6^, ^9^, ^11^, ^18^, ^33^ CN and AD groups were balanced for age and sex. The CN group had a slightly higher education level, which was not significant. CSF sampling was performed an average of 1.5 ± 6.0 months after [^11^C]UCB‐J PET with a range of 12.8 months before to 18.1 months after the PET scan. As expected, the AD group had lower MMSE scores and higher CDR‐global scores. Participants with AD had [^11^C]PiB scans that were positive for brain Aβ, and CN participants were negative for brain Aβ. CSF pTau181 was higher in all participants with AD compared to the CN group. As in our previous analyses of a largely overlapping sample, SV2A binding in both a composite of AD‐affected regions, as well as in the hippocampus, was significantly lower in the AD group.

**TABLE 1:** Participant characteristics.

Parameter	Cognitively normal	Alzheimer's disease	*p*‐Value
Participants (*n*)	7	21 (mild dementia: 17, MCI: 4)	–
Sex (M/F)	5/2	10/11	0.27
Age (years)	72.7 (7.2) (60–81)	69.0 (8.4) (50–84)	0.30
Education (years)	17.1 (2.5) (12–20)	15.6 (2.3) (12–20)	0.15
CDR‐global	0 (0) (0)	0.8 (0.2) (0.5–1.0)	<0.00001
CDR‐SB	0 (0) (0)	4.7 (1.4) (2.5–8.0)	<0.00001
MMSE	28.9 (1.3) (27–30)	23.0 (2.9) (18–29)	0.00003
Amyloid +/‐	0/7	21/0	<0.00001
p‐tau (pg/mL)	27.8 (11.2) (11.4–42.2)	123.0 (77.5) (51.4–347.2)	0.003
Composite SV2A binding (*DVR*)	1.55 (0.08) (1.43–1.67)	1.46 (0.10) (1.31–1.64)	0.04
Hippocampal SV2A binding (*DVR*)	0.99 (0.08) (0.86–1.08)	0.88 (0.12) (0.73–1.17)	0.03
***APOE* ε4 copy number (*n*)**			
2 copies	0	6 (28.6%)	–
1 copy	3 (42.7%)	9 (42.9%)	–
0 copies	4 (57.1%)	6 (28.6%)	–

*Note*: Data are mean (SD) (range). *p*‐values are for unpaired *t*‐tests (continuous variables) or χ^2^ (categorical variables).

Abbreviations: *APOE*, apolipoprotein E; CDR‐global, clinical dementia rating global score; CDR‐SB, Clinical Dementia Rating Scale Sum of Boxes; *DVR*, distribution volume ratio of [^11^C]UCB‐J calculated with a whole cerebellum reference region; MMSE, Mini‐Mental State Examination; SV2A, synaptic vesicle glycoprotein 2A.

### 3.3 Relationship between PET and CSF measurements of SV2A

**FIGURE 1:**
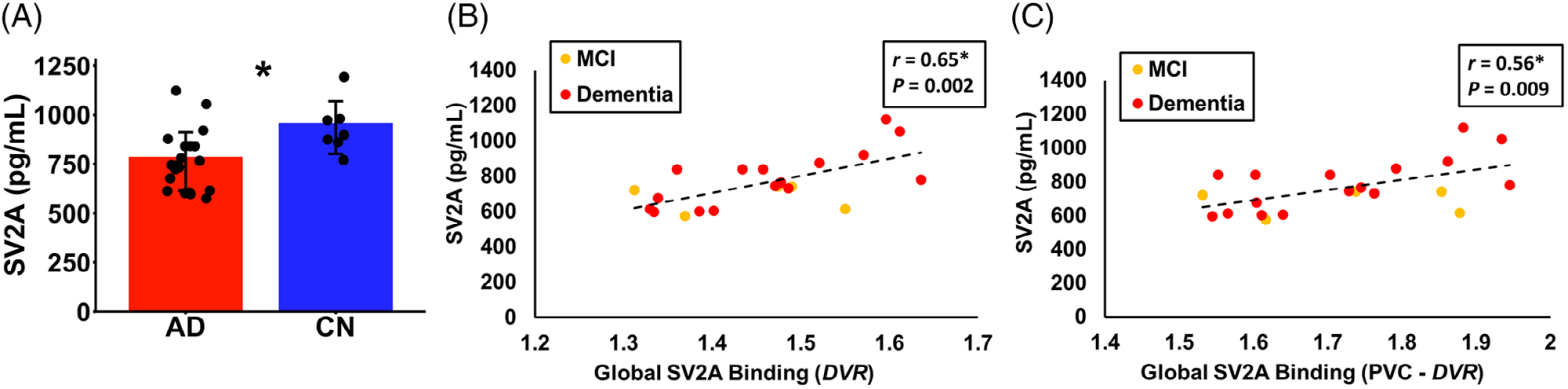
CSF and PET measures of SV2A. (A) SV2A concentration was measured in the CSF for individuals with AD (*n* = 21) and CN (*n* = 7). Dots represent the CSF protein concentration for each participant, and error bars represent the standard deviation. *p < 0.05 for unpaired t‐tests comparing AD to CN. Global SV2A binding (*DVR*) calculated without (B) and with PVC (C) was plotted with CSF SV2A concentration in the group of participants with AD. The disease stage of MCI is represented by yellow dots, and dementia is represented by red dots. Data points representing individual participants and a regression line (dotted) are plotted. *p < 0.05 for the corresponding Pearson's *r*. AD, Alzheimer's disease; CN, cognitively normal; CSF, cerebrospinal fluid; *DVR*, distribution volume ratio of [11C]UCB‐J calculated with a whole cerebellum reference region; MCI, mild cognitive impairment; PET, positron emission tomography; SV2A, synaptic vesicle glycoprotein 2A.

An initial analysis investigated the group differences in CSF SV2A concentrations between the AD and CN groups (Figure 1A). CSF SV2A was significantly lower in participants with AD (762 ± 148 pg/mL, *n* = 21) compared to CN participants (935 ± 134 pg/mL, *n* = 7, unpaired *t*‐test, *p* = 0.01). The primary analysis investigated the association between global SV2A binding by [^11^C]UCB‐J (*DVR*) in a composite of AD‐affected regions and CSF SV2A within the AD group. PET and CSF measurements of SV2A were positively associated in participants with AD (*r* = 0.65, *p* = 0.002, Figure 1B). After PVC of [^11^C]UCB‐J PET images to account for potential bias related to atrophy, this association remained strongly positive (*r* = 0.56, *p* = 0.009, Figure 1C). Sensitivity analyses that included time between [^11^C]UCB‐J PET and CSF collection were also significant (before PVC: *R* = 0.65, *p* = 0.007; after PVC: *R* = 0.56, *p* = 0.03) and SV2A binding was a significant predictor of CSF SV2A within the AD group (before PVC: *η* = 0.64, *p* = 0.002; after PVC: *η* = 0.55, *p* = 0.01).

**FIGURE 2:**
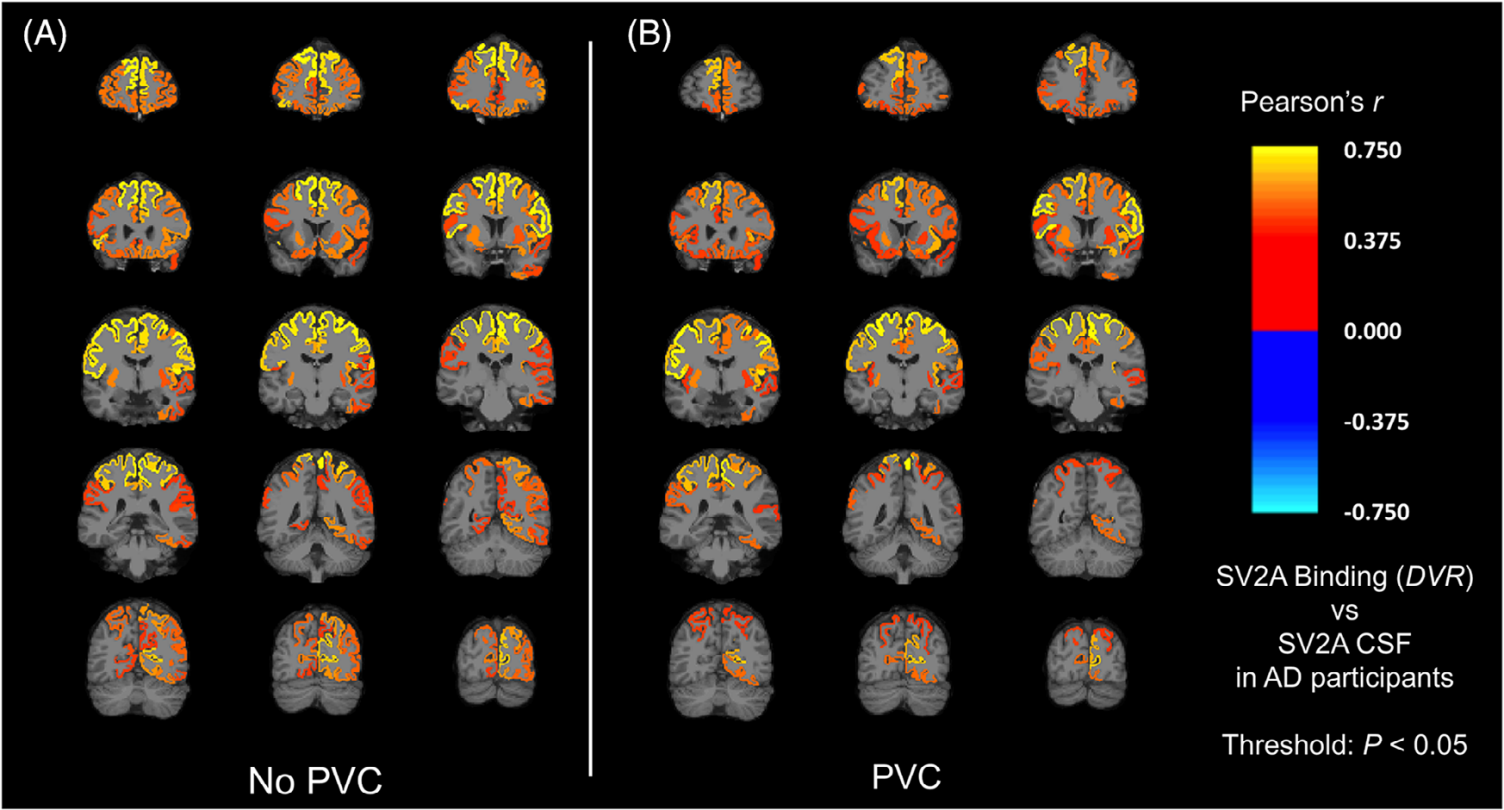
Regional correlations of SV2A binding (*DVR)* and CSF SV2A in AD. (A) Pearson's *r* was calculated for the correlation between SV2A binding and the CSF SV2A concentration for all brain regions in participants with AD (*n* = 21). A similar analysis between SV2A binding and CSF SV2A concentration was conducted (B) after PVC of [^11^C]UCB‐J PET images. Brain maps were created by producing images with the voxels in each brain region set uniformly to the calculated Pearson's *r* for that region and overlaid on an MNI template T1 MRI. The color scale represents Pearson's *r* for regions that had an uncorrected *p *< 0.05. AD, Alzheimer's disease; CSF, cerebrospinal fluid; *DVR*, distribution volume ratio of [^11^C]UCB‐J calculated with a whole cerebellum reference region; MNI, Montreal Neurological Institute; PVC, partial volume correction; PET, positron emission tomography; SV2A, synaptic vesicle glycoprotein 2A.

Exploratory analyses assessed the association between SV2A binding in all brain regions and CSF SV2A. Pearson's *r* was calculated for the correlation between PET and CSF measurements of SV2A (Figure 2A, eTable 4). SV2A binding in many frontal, temporal, parietal, and occipital cortical regions had a significant positive association with CSF SV2A, with a notable lack of significant association in medial temporal regions. A similar pattern of significant, but weaker, associations was seen after PVC of the [^11^C]UCB‐J PET images (Figure 2B, eTable 4).

### 3.4 Relationship between SV2A PET and other CSF biomarkers of synaptic and axonal integrity

**FIGURE 3:**
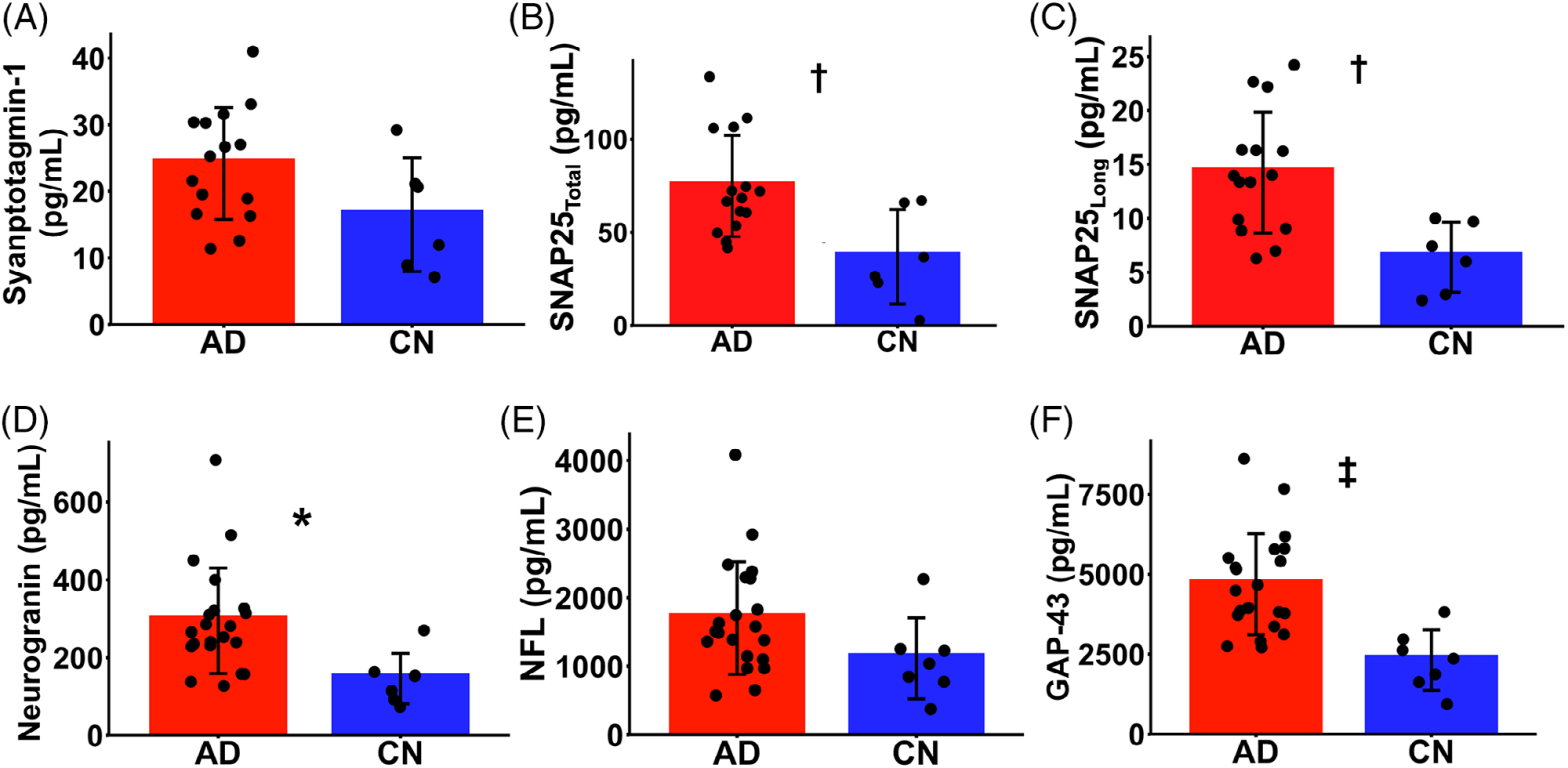
CSF biomarkers of synaptic and neuronal damage in participants with AD and normal cognition. (A) Synaptotagmin‐1 (CN *n* = 6; AD *n* = 15), (B) SNAP25_Total_ (CN *n* = 6; AD *n* = 15), (C) SNAP25_Long_ (CN *n* = 6; AD *n* = 15), (D) Neurogranin (CN *n* = 7; AD *n* = 21), (D) NFL (CN *n* = 7; AD *n* = 21), and (E) GAP‐43 (CN *n* = 7; AD *n* = 21) was measured in the CSF of a group of individuals with AD and a CN group. Dots represent the CSF protein concentration for each participant, and error bars represent the standard deviation. *p < 0.05, ^†^ *p* < 0.01, ‡*p* < 0.001 for unpaired *t‐*tests comparing AD to CN. AD, Alzheimer's disease; CN, cognitively normal; CSF, cerebrospinal fluid; GAP‐43, growth‐associated protein 43; NFL, neurofilament light chain; SNAP25, synaptosomal‐associated protein‐25 kDa.

We also investigated group differences in other CSF biomarkers of synaptic and axonal integrity between the AD and CN groups (Figure 3). All CSF markers measured were higher in participants with AD compared to CN participants, with group differences in SNAP25_Total_, SNAP25_Long_, neurogranin, and GAP‐43 achieving significance (Figure 3, eTable 5).

**FIGURE 4:**
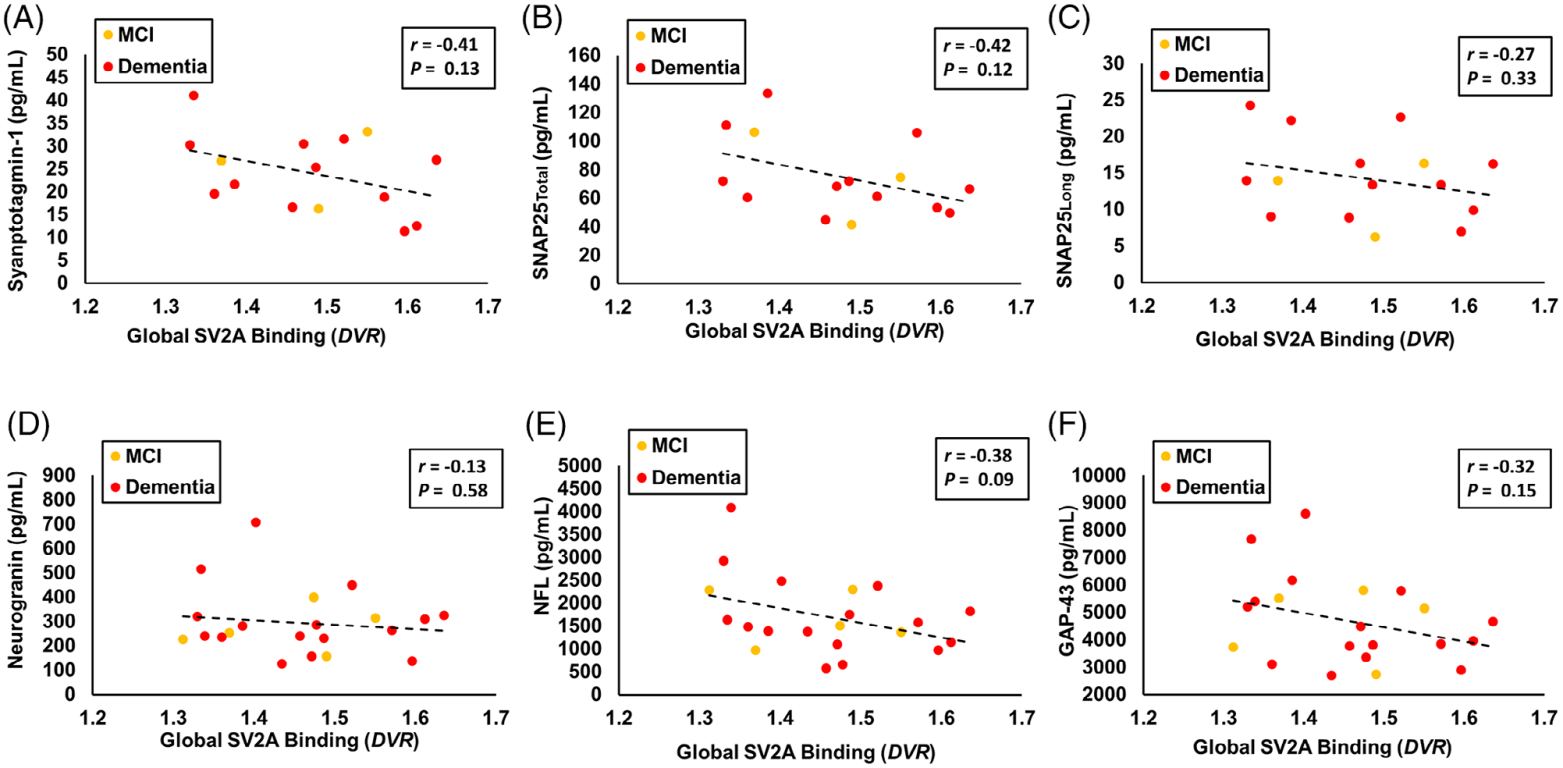
Correlation of SV2A binding (*DVR*) and CSF measures of synaptic or neuronal damage in participants with AD. Global SV2A binding (*DVR*) was plotted with (A) Synaptotagmin‐1 (*n* = 15), (B) SNAP25_Total_ (*n* = 15), (C) SNAP25_Long_ (*n* = 15), (D) Neurogranin (*n* = 21), (E) NFL (*n* = 21), and (F) GAP‐43 (*n* = 21) concentration in the group of participants with AD. The disease stage of MCI is represented by yellow dots, and dementia is represented by red dots. Data points for individual participants and a regression line (dotted) are plotted. *r* and *p*‐values are displayed for Pearson's correlations. AD, Alzheimer's disease; CN, cognitively normal; CSF, cerebrospinal fluid; *DVR*, distribution volume ratio of [^11^C]UCB‐J calculated with a whole cerebellum reference region; GAP‐43, growth‐associated protein 43; MCI, mild cognitive impairment; synaptic vesicle glycoprotein 2A; NFL, neurofilament light chain; SNAP25, synaptosomal‐associated protein‐25 kDa; SV2A, synaptic vesicle glycoprotein 2A.

Additional analyses assessed the associations between global SV2A binding (*DVR*) in a composite of AD‐affected regions and CSF biomarkers within the AD group (Figure 4). Nonsignificant, weak to moderate strength, negative associations were found with the strongest trends for association between global SV2A binding and synaptotagmin‐1 (*r* = −0.41, *p* = 0.13, Figure 4A), SNAP_Total_ (*r* = −0.42, *p* = 0.12, Figure 4B), NFL (*r* = −0.38, *p* = 0.09, Figure 4E), and GAP‐43 (*r* = −0.32, *p* = 0.15, Figure 4F). After PVC of the [^11^C]UCB‐J PET images, these associations were weak and not significant (eFigure 3).

### 3.5 Associations between SV2A binding (*DVR*) in all brain regions and CSF measures of synaptic or axonal integrity in participants with AD

Further exploratory analyses assessed the association between SV2A binding in all brain regions and CSF biomarkers, including synaptotgmin‐1, SNAP25_Total_, SNAP25_Long_, neurogranin, NFL, and GAP‐43 (eFigure 4). Synaptotgmin‐1 was negatively associated with SV2A binding in the left fusiform gyrus, left paracentral gyrus, left precentral gyrus, right superior frontal gyrus, right pars orbitalis, right pars opercularis, right paracentral gyrus, right postcentral gyrus, and right precentral gyrus (eFigure 4, eTable 6). SNAP25_Total_ was negatively associated with SV2A binding in the left frontal pole, left superior frontal gyrus, left temporal pole, left entorhinal cortex, left parahippocampal cortex, left rostral anterior cingulate, left caudal anterior cingulate, left postcentral gyrus, left lingual gyrus, left nucleus accumbens, right superior frontal gyrus, right lateral orbitofrontal cortex, right medial orbitofrontal cortex, right temporal pole, right amygdala, right caudal anterior cingulate, and right precentral gyrus (eFigure 4, eTable 7). SNAP25_Long_ was negatively associated with SV2A binding in only the right postcentral gyrus and right precentral gyrus (eFigure 4, eTable 8). Neurogranin was positively associated with SV2A binding in the left amygdala, left pallidum, right hippocampus, and right pallidum (eFigure 4, eTable 9). NFL was negatively associated with SV2A binding in the right superior frontal gyrus, right pars triangularis, right parahippocampal cortex, right transverse temporal gyrus, right supramarginal gyrus, right postcentral gyrus, right precentral gyrus, and right cuneus (eFigure 4, eTable 10). GAP‐43 was negatively associated with SV2A binding in the left paracentral gyrus, left cuneus, left lingual gyrus, right pars orbitalis, and right paracentral gyrus (eFigure 4, eTable 11).

After PVC, similar patterns persisted for each CSF biomarker, but with generally weaker associations (eFigure 5, eTables 6–11). One exception was for synaptotagmin‐1, where a stronger association was seen after PVC in the right temporal pole, right nucleus accumbens, and right ventral diencephalon (eFigure 5, eTable 6).

## 4 DISCUSSION

In this study, we examined the relationship between synaptic density as measured by SV2A PET ([^11^C]UCB‐J *DVR*) and CSF biomarkers reflecting synaptic and axonal integrity. Our primary analysis focused on a novel CSF measure of SV2A, the presynaptic protein that is also the target of the PET radioligand [^11^C]UCB‐J. CSF SV2A concentration was significantly lower in individuals with AD compared to CN participants, and within the AD group, global SV2A binding was significantly positively correlated with CSF SV2A. Exploratory regional analyses suggested that [^11^C]UCB‐J PET measurement of SV2A may be associated with CSF SV2A most strongly in neocortical regions but not in medial temporal regions. Other CSF synaptic and axonal proteins were generally higher in participants with AD, with significant differences in SNAP25Total, SNAP25Long, Ng, synaptotagmin‐1, and GAP‐43. However, none of these proteins was significantly associated with SV2A concentrations measured with PET. Exploratory analyses of all brain regions suggested that SV2A binding in specific regions may be associated with some CSF biomarkers.

The high degree of association between CSF and PET measurements of SV2A in individuals with AD and the significant reductions in AD compared to CN participants provides support for a novel CSF assay for SV2A that may serve as an additional biomarker for synaptic density in AD. These findings further suggest that CSF measurement of SV2A may be a reasonable secondary outcome in longitudinal observational studies and therapeutic trials in AD. The fact that the association with CSF SV2A was strongest for PET measurements in neocortical regions but not medial temporal regions (eTable 4) is interesting but may reflect the greater contribution of large neocortical regions to CSF levels of SV2A. The prodromal MCI stage of AD appears to involve an already robust SV2A reduction in medial temporal lobe regions than in neocortical regions, with only small differences in medial temporal SV2A between MCI and AD dementia (see Mecca et al.; Supplementary Figure 2, Supplementary Table S4B).^7^ By contrast, neocortical regions tend to have losses across the AD continuum, which are numerically greater in dementia than MCI (see Mecca et al.; Supplementary Figure 2, Supplementary Table S4B).^7^ Thus, PET measures in the larger neocortical regions may correlate better with CSF SV2A, whereas PET measures in medial temporal lobe may reach a relative “floor” at an earlier stage and thus not be significantly associated with CSF SV2A within the AD group.

In a companion study (Nilsson et al.,)^35^, we examined the relationship between [^11^C]UCB‐J PET and some additional fluid biomarkers of synaptic pathology using a mass spectrometric synaptic protein panel. Within the AD group, the most robust global associations were seen for syntaxin‐7 and phosphatidylethanolamine binding protein 1 (PEBP‐1). However, PEBP‐1 displayed higher levels in the AD compared to the CN group, whereas syntaxin‐1B and syntaxin‐7 (trend‐level) showed higher values in the AD group.

Overall, these two studies suggest that in the setting of AD, CSF SV2A has a relationship with global [^11^C]UCB‐J PET measures of SV2A that is opposite to that of most of the other synaptic markers evaluated. CSF SV2A shows *lower* values in AD compared to controls and *positive* associations with SV2A PET in the AD group. The other synaptic markers, including four other synaptic vesicle proteins (synaptotagmin‐1, SNAP25, syntaxin‐1B, and syntaxin‐7), generally show the reverse pattern, with higher values in AD for synaptotagmin‐1, SNAP25, syntaxin‐1B, and syntaxin‐7 (trend‐level). These markers also generally show negative associations with SV2A PET in the AD group, which is significant for syntaxin‐1B, syntaxin‐7, and at a trend‐level for synaptotagmin‐1 and SNAP25. The reason for the different pattern with CSF SV2A is unclear, but it may indicate that SV2A in CSF in AD is largely present as part of normal physiology, and its concentrations thus decline with synaptic loss and are not increased by the neurodegenerative changes of AD. Conversely, the other proteins may appear in CSF in AD, largely related to neurodegeneration, with a resultant negative association with synaptic density as assessed by PET. Why SV2A would show a pattern that is opposite to that of the other proteins is unclear since SV2A—like synaptotagmin—is localized to synaptic vesicles as a transmembrane protein^36^; whereas SNAP25 is anchored to the cytosolic face of membranes,^37^ and syntaxin is localized to the plasma membrane as a transmembrane protein.^38^

This study has a number of important limitations. The analysis is cross‐sectional and suggests that changes in CSF biomarkers are related to synaptic loss, but longitudinal studies are needed to fully explore the longitudinal relationships between PET and CSF biomarkers of synaptic and axonal integrity. Although we are able to detect statistically significant relationships between PET and CSF measures of SV2A, the sample size is relatively small with limited power to assess the weaker relationships that may exist between SV2A PET and other CSF biomarkers. To illustrate, the next largest effect size (Pearson's *r*) after SV2A PET and CSF SV2A is for SV2A PET and SNAP25_Long_ (*r* = −0.42), where a sample size of *n* = 24 would be needed to achieve *p* < 0.05. While the consistency of relationships both with and without application of PVC increases our confidence of the results, effects sizes are generally smaller with PVC applied. This reduced effect size may indicate that, in AD, apparent tracer uptake is influenced by both gray matter atrophy and SV2A density in the remaining tissue. However, the reduced effect size may also be due to errors in the assumptions of the PVC algorithm and additional measurement error introduced by PVC methods, as assessed and reviewed in our previous publication.^39^ CSF sampling was performed an average of 1.5 months (range of 12.8 months before to 18.1 months after) after the PET scan. Performing measurements closer in time would likely increase our ability to detect associated changes in biomarkers. Future studies should also explore fluid biomarker relationships with an18F‐labeled SV2A PET tracer that will be more accessible for larger, multisite studies. Finally, we have focused on individuals with early symptomatic AD, but additional studies are needed to understand the relationships between SV2A PET and CSF biomarkers in CN participants, including those with confirmed underlying AD pathogenesis.

## 5 CONCLUSION

This study reports a novel CSF assay for SV2A that is significantly lower in individuals with AD compared to CN participants, and within the AD group, is strongly correlated with global [^11^C]UCB‐J PET measures of SV2A. Exploratory regional analyses suggested that PET measurement of SV2A may be associated with CSF SV2A most strongly in neocortical regions but not in medial temporal regions. In contrast to SV2A, other CSF proteins were generally numerically higher in participants with AD and had only weak associations with SV2A PET. The results suggest that CSF SV2A is a biomarker for synaptic density in AD and support the continued development of fluid‐based biomarkers of synaptic health.

## CONFLICT OF INTEREST STATEMENT

A.P.M., R.E.C., and C.H.vD. report grants from National Institutes of Health for the conduct of the study. A.P.M. reports grants for clinical trials from Genentech, Janssen, and Eli Lilly outside the submitted work. M.K.C. reports grants for clinical trials from Merck outside the submitted work. Y.H. reports research grants from the UCB and Eli Lilly outside the submitted work. Y.H., R.E.C., and N.B.N. have a patent for a newer version of the tracer. REC reports having received grants from Bristol Myers Squibb, Cerevel Therapeutics, outside the submitted work. C.H.vD. reports consulting fees from Kyowa Kirin, Roche, Merck, Eli Lilly, and Janssen and grants for clinical trials from Biogen, Novartis, Eli Lilly, Merck, Eisai, Janssen, Roche, Genentech, Toyama, and Biohaven, outside the submitted work. O.H. is an employee of Eli Lilly and Lund University, and he has previously acquired research support (for Lund University) from AVID Radiopharmaceuticals, Biogen, C2N Diagnostics, Eli Lilly, Eisai, Fujirebio, GE Healthcare, and Roche. He has received consultancy/speaker fees from Alzpath, BioArctic, Biogen, Bristol Meyer Squibb, Eisai, Eli Lilly, Fujirebio, Merck, Novartis, Novo Nordisk, Roche, Sanofi and Siemens. H.Z. has served at scientific advisory boards and/or as a consultant for Abbvie, Acumen, Alector, Alzinova, ALZpath, Amylyx, Annexon, Apellis, Artery Therapeutics, AZTherapies, Cognito Therapeutics, CogRx, Denali, Eisai, Enigma, LabCorp, Merry Life, Nervgen, Novo Nordisk, Optoceutics, Passage Bio, Pinteon Therapeutics, Prothena, Quanterix, Red Abbey Labs, reMYND, Roche, Samumed, Siemens Healthineers, Triplet Therapeutics, and Wave, has given lectures sponsored by Alzecure, BioArctic, Biogen, Cellectricon, Fujirebio, Eli Lilly, Novo Nordisk, Roche, and WebMD, and is a co‐founder of Brain Biomarker Solutions in Gothenburg AB (BBS), which is a part of the GU Ventures Incubator Program (outside submitted work). K.B. has served as a consultant and at advisory boards for Abbvie, AC Immune, ALZPath, AriBio, BioArctic, Biogen, Eisai, Eli Lilly, Moleac Pte. Ltd, Neurimmune, Novartis, Ono Pharma, Prothena, Roche Diagnostics, Sanofi and Siemens Healthineers; has served at data monitoring committees for Julius Clinical and Novartis; has given lectures, produced educational materials and participated in educational programs for AC Immune, Biogen, Celdara Medical, Eisai and Roche Diagnostics; and is a co‐founder of Brain Biomarker Solutions in Gothenburg AB (BBS), which is a part of the GU Ventures Incubator Program, outside the work presented in this paper. No other disclosures are reported. Author disclosures are available in the Supporting Information.

## CONSENT STATEMENT

All participants provided informed consent. The study was approved by the Yale Human Investigations Committee and was performed in accordance with the ethical standards as laid down in the 1964 Declaration of Helsinki and its later amendments or comparable ethical standards.

## Supporting information



Supporting Information

Supporting Information
