# Supplementary material for: Cerebrospinal fluid and brain positron emission tomography measures of synaptic vesicle glycoprotein 2A: Biomarkers of synaptic density in Alzheimer's disease
Source: Alzheimers Dement. 2025 Jun 9;21(6):e70344. doi: 10.1002/alz.70344 (PMC12149441; doi:10.1002/alz.70344)
Supplement: Supplementary file 1 — Supporting Information [file ALZ-21-e70344-s001.docx]

**Supplementary Materials**

1. **eMethods**
2. **eTables**
3. **eFigures**
4. **References**
5. **eMethods**

***1.1 Study Participants and Design***

All participants in the primary analysis scan cohort were recruited at Yale University and received a Positron Emission Tomography (PET) with [^11^C]Pittsburgh Compound B ([^11^C]PiB) to determine the presence of brain amyloid-β accumulation. The [^11^C]PiB PET scan was considered positive if both visual and quantitative criteria were met. Visual reading was performed by an experienced readers (APM, MKC or RSO) and quantitative criteria required a [^11^C]PiB cerebral-to-cerebellar distribution volume ratio (*DVR*) of 1.40 or more in at least one AD-affected region of interest (ROI) [1, 2]. *APOE* genotyping was also performed as previously described [2].

Participants from cerebrospinal fluid (CSF) discovery cohort 1 and discovery cohort 2 were recruited from Sahlgrenska University Hospital and Memory Clinic at the University of Lund, respectively. Discovery cohort 1 included patients with Alzheimer’s disease (AD) (n = 20) and typical AD CSF biomarkers profiles (Aβ1-42 < 530 ng/L, p-tau181 > 60 ng/L, and t-tau > 350 ng/L), and cognitively unimpaired control participants (CU, n = 20) who were patients examined at the memory or neurology clinics for minor neurological or psychiatric symptoms with basic and core CSF biomarkers levels within normal ranges. Discovery cohort 2 included patients with AD (n = 31) and CU control participants (n = 56). The applied IWG-2 criterion[3] for allocation to the AD group was a low concentration of Aβ42 (< 550 ng/L) with a high level of t-tau (> 400 ng/L) or p-tau181 (> 80 ng/L). CU controls with Aβ42 < 550 ng/L were classified as CU Aβ-positive (CU Aβ+). All CU participants had tau biomarkers within normal ranges.

***1.2. Magnetic Resonance Imaging***

Magnetic Resonance Imaging (MRI) was performed on a 3T Trio (Siemens Medical Systems, Erlangen, Germany) with a circularly polarized head coil. MR acquisition consisted of a Sag 3D magnetization-prepared rapid gradient-echo (MPRAGE) sequence with 3.34-msec echo time, 2500-msec repetition time, 1100-msec inversion time, 7º flip angle, and 180 Hz/pixel bandwidth. Images are 256x256x176 with a pixel size of 0.98x0.98x1.0 mm. The MRI ensured that patients did not show evidence of infection, infarction, or other brain lesions. In addition, the MRI was used to define anatomy and to perform partial volume correction (PVC) [4, 5] as described in our previous analyses [6].

***1.3 Positron Emission Tomography***

PET scans were performed on the HRRT (207 slices, resolution <3 mm FWHM) [7]. List-mode data were reconstructed using the MOLAR algorithm [8] with event-by-event motion correction based on an optical detector (Vicra, NDI Systems, Waterloo, Canada) [9]. Dynamic [^11^C]PiB scans were acquired for 90 min following administration of a bolus of up to 555 MBq of tracer [2]. Dynamic [^11^C]UCB-J scans were acquired for up to 90 min after administration of a bolus of up to 740 MBq [10]. Software motion correction was applied to the dynamic PET images using a mutual-information algorithm (FSL-FLIRT) to perform frame-by-frame registration to a summed image (0-10min). A summed motion corrected PET image was registered to the participant’s MRI. For each participant’s MRI, cortical reconstruction and volumetric segmentation was performed using FreeSurfer [version 6.0, http://surfer.nmr.mhg.harvard.edu/] [11]. ROIs from the FreeSurfer segmentation were used for both PET and MRI analyses performed in native subject space. PVC was performed using the Iterative Yang approach[4, 5] prior to kinetic modeling.

***1.4. Tracer Kinetic Modeling***

Tracer kinetic modeling was performed as detailed in our previous study [12]. For [^11^C]PiB image analysis, parametric images of *BP*_ND_ (the ratio at equilibrium of specifically bound radioligand to that of nondisplaceable radioligand in tissue) [13] were generated using SRTM2 [14] with whole cerebellum as reference region. *BP*_ND_ was calculated so that a value of 0 reflects no specific binding, i.e., tracer uptake no greater than that in the reference region. This is directly related to the *DVR* reported by other investigators [1], in that *DVR*=*BP*_ND_+1.

For [^11^C]UCB-J, we generated parametric images of *BP*_ND_ using a simplified reference tissue model – 2 step (SRTM2) [14] and a small ROI (2 mL) in the core of the centrum semiovale (CS) as reference region [15, 16]. The use of SRTM2 requires a global $k_{2}^{'}$ value (clearance rate constant, *k*_2_, of the reference region), which was computed as a population average of *k*_2_ of the CS obtained using the 1TC model ($k_{2}^{'}$=0.027 min^-1^; from a previous group of subjects with arterial blood sampling) [12, 17]. As previously described, *DVR* values using a whole cerebellum reference region were computed for each voxel as (*BP*_ND_ +1)/(*BP*_ND_[cerebellum]+1) [12].

***1.5. Cerebrospinal fluid Synaptic vesicle glycoprotein 2A (SV2A) enzyme-linked immunosorbent assay (ELISA)***

Nunc-Immuno Polysorp microwell modules (Thermo Fisher Scientific, Massachusetts, US) were coated with mouse anti-SV2A antibody (62.5 ng/ml) in carbonate buffer pH 9.6, overnight at 4°C. After washing, wells were blocked with 5% bovine serum albumin in assay diluent for 1h at room temperature. Thereafter, in house recombinant N-terminal SV2A calibrators in 0.5% n-octylglucopyranoside (316 pg/ml - 20000 pg/ml), blanks, internal control samples and CSF samples were incubated at room temperature for 5h. After washing, a rabbit detector antibody (0.13µg/ml) was applied and incubated overnight at 4°C. After additional washes, plates were incubated with anti-rabbit HRP (1:20000) for 2.5h. After subsequent washes, wells were incubated for 20 minutes with 3,3´,5,5´- tetramethylbenzidine (TMB, KemEnTech Diagnostics) in dark. The colour reaction was stopped by addition of 0.2 M H2SO4 and the absorbance was read in a SunriseTM microplate absorbance reader (Tecan group, Männedorf, Switzerland) at 450 nm (650 nm as reference value). CSF sample concentration was calculated via interpolation from the calibrator curve (4PL weighted 1/Y2).

***1.6. Cerebrospinal fluid SV2A ELISA validation***

The performance of the novel SV2A ELISA was validated using deanonymized CSF samples from clinical routine of the Neurochemistry laboratory, Sahlgrenska University Hospital, Mölndal, Sweden. The assay validation focused on intermediate precision, parallelism, and dilution linearity in three CSF pools determined to be high (2112 pg/mL), intermediate (1456 pg/mL), and low (750 pg/mL) in SV2A levels. The assay intermediate precision (14.5%) was determined by measuring the SV2A concentration in the three CSF pools, aliquoted, and stored at -80°C, in 5-7 duplicates on three different occasions. For parallelism, CSF samples were analyzed undiluted or diluted (2-fold and 4-fold) and the % recovery was calculated (eTable 10). Dilution linearity (eTable 11) was measured by spiking in a known concentration of recombinant SV2A (XX.X pg/mL) into the three CSF samples and quantifying undiluted or diluted (2-fold and 4-fold). The lower limit of quantification (325 pg/mL) was calculated as the mean of 12 blank duplicates plus 10 times its standard deviation.

**2. eTables**

| **eTable 1. Individual regions included in the composite of AD-affected regions.** | | | |
| --- | --- | --- | --- |
| **General Region** | **Freesurfer Region** | **Right Label Number** | **Left Label Number** |
| **Prefrontal** | Frontal pole | 2032 | 1032 |
|  | Superior frontal gyrus | 2028 | 1028 |
|  | Rostral middle frontal gyrus | 2027 | 1027 |
|  | Caudal middle frontal gyrus | 2003 | 1003 |
|  | Pars orbitalis | 2019 | 1019 |
|  | Pars opercularis | 2018 | 1018 |
|  | Pars triangularis | 2020 | 1020 |
|  | Lateral orbitofrontal cortex | 2012 | 1012 |
|  | Medial orbitofrontal cortex | 2014 | 1014 |
| **Anterior Cingulum** | Rostral Anterior Cingulate Cortex | 2026 | 10026 |
|  | Caudal Anterior Cingulate Cortex | 2002 | 1002 |
| **Lateral temporal** | Temporal pole | 2033 | 1033 |
|  | Inferior temporal gyrus | 2009 | 1009 |
|  | Fusiform gyrus | 2007 | 1007 |
|  | Middle temporal gyrus | 2015 | 1015 |
|  | Banks of the superior temporal sulcus | 2001 | 1001 |
|  | Superior temporal gyrus | 2030 | 1030 |
|  | Transverse temporal gyrus | 2034 | 1034 |
| **PCC/Precuneus** | Posterior cingulate cortex | 2023 | 1023 |
|  | Isthmus of the cingulum | 2010 | 1010 |
|  | Precuneus | 2025 | 1025 |
| **Lateral parietal** | Superior parietal lobule | 2029 | 1029 |
|  | Inferior parietal lobule | 2008 | 1008 |
|  | Supramarginal gyrus | 2031 | 1031 |
| **Lateral Occipital** | Lateral occipital cortex | 2011 | 1011 |

Label names and numbers are from the FreeSurfer [Version 6.0] Desikan-Killiany atlas and look up table for label numbers. All regions above were used to construct a composite of common Alzheimer’s disease (AD) – affected regions. Composite regions are volume-weighted averages of the subregions.

**eTable 2. Validation of CSF SV2A immunoassay – Parallelism**

| Sample Number | Fold Dilution | Mean calculated concentration (pg/mL) | Concentration CV (%) | Dilution corrected concentration (pg/mL) | Recovery (%) |
| --- | --- | --- | --- | --- | --- |
| 1 – high CSF SV2A | 1 | 2112 | 12.1 | 2112 |  |
|  | 2 | 1235 | 13.2 | 2470 | 117 |
|  | 4 | 673 | 15.2 | 2692 | 127 |
|  | 8 | 398 | 17.8 | 3184 | 150 |
| 2 – intermediate CSF SV2A | 1 | 1456 | 10.9 | 1456 |  |
|  | 2 | 804 | 12.5 | 1608 | 110 |
|  | 4 | 475 | 17.1 | 1900 | 130 |
|  | 8 | < LLOQ | | | |
| 3 – low CSF SV2A | 1 | 750 | 15.2 | 750 |  |
|  | 2 | 450 | 16.3 | 900 | 120 |
|  | 4 | < LLOQ | | | |
|  | 8 | < LLOQ | | | |

Abbreviations: CSF, cerebrospinal fluid; CV, coefficient of variation; SV2A, synaptic vesicle glycoprotein 2A

**eTable 3. Validation of CSF SV2A immunoassay – Dilution linearity**

| Sample Number | Fold Dilution | Expected (pg/mL) | Observed (pg/mL) | Recovery (%) |
| --- | --- | --- | --- | --- |
| 1 – 2122 pg/mL | 1 | 3122 | 2871 | 91.9% |
|  | 2 | 1561 | 1871 | 119% |
|  | 4 | 780.5 | 922 | 118% |
|  | 8 | 390.25 | 522 | 133% |
| 2 – 1456 pg/mL | 1 | 1946 | 1872 | 96.2% |
|  | 2 | 973 | 1035 | 106.5% |
|  | 4 | 486.5 | 567 | 116% |
|  | 8 | 243.25 | 370 | 152% |

^*^Expected concentration = sample SV2A pg/mL + spike concentration. Abbreviations: CSF, cerebrospinal fluid; SV2A, synaptic vesicle glycoprotein 2A

| **eTable 4. Correlations between SV2A PET (*DVR*) and CSF SV2A in participants with AD** | | | | | |
| --- | --- | --- | --- | --- | --- |
|  | ***DVR*** | | **PVC *– DVR*** | | |
| **Left Hemisphere Region** | **Pearson’s *r*** | ***P*** | | **Pearson’s *r*** | ***P*** |
| Frontal pole | 0.53 | 0.01* | | 0.19 | 0.41 |
| Superior frontal gyrus | 0.71 | 0.0004* | | 0.55 | 0.01* |
| Rostral middle frontal gyrus | 0.53 | 0.01* | | 0.26 | 0.26 |
| Caudal middle frontal gyrus | 0.57 | 0.01* | | 0.54 | 0.01* |
| Pars orbitalis | 0.36 | 0.11 | | 0.19 | 0.41 |
| Pars opercularis | 0.53 | 0.01* | | 0.50 | 0.02* |
| Pars triangularis | 0.59 | 0.005* | | 0.52 | 0.02* |
| Lateral orbitofrontal cortex | 0.57 | 0.01* | | 0.50 | 0.02* |
| Medial orbitofrontal cortex | 0.57 | 0.01* | | 0.54 | 0.01* |
| Temporal pole | 0.38 | 0.09 | | 0.34 | 0.13 |
| Entorhinal cortex | 0.32 | 0.16 | | 0.34 | 0.13 |
| Parahippocampal cortex | 0.31 | 0.17 | | 0.29 | 0.20 |
| Hippocampus | 0.18 | 0.42 | | 0.19 | 0.40 |
| Amygdala | 0.27 | 0.24 | | 0.31 | 0.16 |
| Inferior temporal gyrus | 0.47 | 0.03* | | 0.39 | 0.08 |
| Fusiform gyrus | 0.56 | 0.01* | | 0.53 | 0.01* |
| Middle temporal gyrus | 0.34 | 0.13 | | 0.27 | 0.25 |
| Banks of the superior temporal sulcus | 0.41 | 0.07 | | 0.24 | 0.30 |
| Superior temporal gyrus | 0.47 | 0.03* | | 0.44 | 0.04* |
| Transverse temporal gyrus | 0.2 | 0.37 | | 0.34 | 0.14 |
| Supramarginal gyrus | 0.45 | 0.04* | | 0.36 | 0.11 |
| Insular cortex | 0.59 | 0.01* | | 0.64 | 0.002* |
| Rostral anterior cingulate cortex | 0.44 | 0.04* | | 0.42 | 0.06* |
| Caudal anterior cingulate cortex | 0.64 | 0.002* | | 0.54 | 0.01* |
| Posterior cingulate cortex | 0.65 | 0.001* | | 0.55 | 0.01* |
| Isthmus of the cingulum | 0.28 | 0.23 | | 0.28 | 0.22 |
| Precuneus | 0.45 | 0.04* | | 0.40 | 0.07 |
| Paracentral gyrus | 0.81 | < 0.0001* | | 0.77 | < 0.0001* |
| Postcentral gyrus | 0.69 | 0.0006* | | 0.58 | 0.01* |
| Precentral gyrus | 0.74 | 0.0001* | | 0.72 | 0.0003* |
| Superior parietal lobule | 0.59 | 0.005* | | 0.45 | 0.04* |
| Inferior parietal lobule | 0.52 | 0.02* | | 0.41 | 0.06 |
| Lateral occipital cortex | 0.52 | 0.02* | | 0.35 | 0.12 |
| Cuneus | 0.67 | 0.0008* | | 0.63 | 0.002* |
| Pericalcarine cortex | 0.68 | 0.0007* | | 0.67 | 0.0008* |
| Lingual gyrus | 0.59 | 0.005* | | 0.53 | 0.01* |
| Thalamus | 0.29 | 0.21 | | 0.34 | 0.13 |
| Caudate | 0.41 | 0.07 | | 0.41 | 0.06 |
| Putamen | 0.50 | 0.02* | | 0.46 | 0.04* |
| Pallidum | -0.06 | 0.80 | | -0.12 | 0.62 |
| Nucleus accumbens | 0.6 | 0.004* | | 0.51 | 0.02* |
| Ventral diencephalon | 0.3 | 0.19 | | 0.33 | 0.15 |
| **eTable 4 Continued.** | | | | | |
|  | ***DVR*** | | **PVC *– DVR*** | | |
| **Right Hemisphere Region** | **Pearson’s *r*** | ***P*** | | **Pearson’s *r*** | ***P*** |
| Frontal pole | 0.42 | 0.06 | | 0.05 | 0.81 |
| Superior frontal gyrus | 0.75 | < 0.0001* | | 0.67 | 0.0009* |
| Rostral middle frontal gyrus | 0.54 | 0.01* | | 0.37 | 0.10 |
| Caudal middle frontal gyrus | 0.53 | 0.01* | | 0.53 | 0.01* |
| Pars orbitalis | 0.68 | 0.0007* | | 0.55 | 0.01* |
| Pars opercularis | 0.47 | 0.03* | | 0.46 | 0.04* |
| Pars triangularis | 0.48 | 0.03* | | 0.49 | 0.03* |
| Lateral orbitofrontal cortex | 0.52 | 0.02* | | 0.46 | 0.04* |
| Medial orbitofrontal cortex | 0.54 | 0.01* | | 0.51 | 0.02* |
| Temporal pole | 0.36 | 0.11 | | 0.35 | 0.12 |
| Entorhinal cortex | -0.2 | 0.38 | | -0.21 | 0.36 |
| Parahippocampal cortex | 0.26 | 0.26 | | 0.28 | 0.22 |
| Hippocampus | -0.05 | 0.83 | | -0.02 | 0.92 |
| Amygdala | 0.2 | 0.40 | | 0.23 | 0.32 |
| Inferior temporal gyrus | 0.27 | 0.24 | | 0.24 | 0.29 |
| Fusiform gyrus | 0.29 | 0.21 | | 0.31 | 0.17 |
| Middle temporal gyrus | 0.17 | 0.47 | | 0.18 | 0.44 |
| Banks of the superior temporal sulcus | 0.35 | 0.12 | | 0.37 | 0.10 |
| Superior temporal gyrus | 0.36 | 0.11 | | 0.42 | 0.06 |
| Transverse temporal gyrus | 0.21 | 0.35 | | 0.30 | 0.18 |
| Supramarginal gyrus | 0.46 | 0.04 | | 0.51 | 0.02* |
| Insular cortex | 0.36 | 0.11 | | 0.45 | 0.04* |
| Rostral anterior cingulate cortex | 0.47 | 0.03 | | 0.49 | 0.02* |
| Caudal anterior cingulate cortex | 0.54 | 0.01 | | 0.45 | 0.04* |
| Posterior cingulate cortex | 0.62 | 0.003 | | 0.52 | 0.02* |
| Isthmus of the cingulum | 0.22 | 0.34 | | 0.25 | 0.28 |
| Precuneus | 0.43 | 0.05 | | 0.43 | 0.05 |
| Paracentral gyrus | 0.72 | 0.0002 | | 0.67 | 0.001* |
| Postcentral gyrus | 0.68 | 0.0008 | | 0.66 | 0.001* |
| Precentral gyrus | 0.72 | 0.0002 | | 0.75 | 0.0001* |
| Superior parietal lobule | 0.52 | 0.02 | | 0.46 | 0.03* |
| Inferior parietal lobule | 0.24 | 0.30 | | 0.27 | 0.23 |
| Lateral occipital cortex | 0.28 | 0.21 | | 0.30 | 0.18 |
| Cuneus | 0.49 | 0.02 | | 0.41 | 0.07 |
| Pericalcarine cortex | 0.56 | 0.01 | | 0.53 | 0.01* |
| Lingual gyrus | 0.46 | 0.04 | | 0.42 | 0.06 |
| Thalamus | 0.04 | 0.85 | | 0.08 | 0.73 |
| Caudate | 0.43 | 0.05 | | 0.43 | 0.05 |
| Putamen | 0.57 | 0.01 | | 0.57 | 0.01* |
| Pallidum | 0.17 | 0.47 | | 0.11 | 0.65 |
| Nucleus accumbens | 0.33 | 0.14 | | 0.36 | 0.11 |
| Ventral diencephalon | 0.16 | 0.50 | | 0.17 | 0.45 |

Pearson’s r and an associated *P*-value (uncorrected for multiple comparisons) are listed. **P* < 0.05 Abbreviations: CSF, cerebrospinal fluid; *DVR*, distribution volume ratio of [^11^C]UCB-J calculated with a cerebellum reference region; AD, Alzheimer’s disease; PET, Positron Emission Tomography; PVC, partial volume correction; SV2A, synaptic vesicle glycoprotein 2A

| **eTable 5. CSF biomarkers of synaptic and neuronal damage** | | |  |
| --- | --- | --- | --- |
|  | **Cognitively Normal** | **Alzheimer's Disease** | ***P*** |
| **Synaptotagmin-1 (pg/mL)** | 16.5 (8.5) | 24.2 (8.4) | 0.01* |
| **SNAP25_Total_ (pg/mL)** | 36.9 (25.3) | 74.8 (27.2) | 0.008‡ |
| **SNAP25_Long_ (pg/mL)** | 6.4 (3.3) | 14.2 (5.6) | 0.005^†^ |
| **Neurogranin (pg/mL)** | 145.9 (64.6) | 294.5 (135.6) | 0.01* |
| **NFL (pg/mL)** | 1112.1 (592.2) | 1702.5 (820.4) | 0.09 |
| **GAP-43 (pg/mL)** | 2314.3 (945.0) | 4688.2 (1573.6) | 0.0009‡ |

Data are mean (SD). **P* < 0.05, ^†^*P* < 0.01, ‡*P* < 0.001 for unpaired *t-*tests comparing AD to CN. Abbreviations: CSF, cerebrospinal fluid; CN, cognitively normal; AD, Alzheimer’s disease; SNAP25, Synaptosomal-Associated Protein-25kDa; NFL, Neurofilament Light Chain; GAP43, Growth Associated Protein 43

| **eTable 6. Correlations between SV2A PET (*DVR*) and CSF synaptotagmin-1 in participants with AD** | | | | | |
| --- | --- | --- | --- | --- | --- |
|  | ***DVR*** | | **PVC *– DVR*** | | |
| **Left Hemisphere Region** | **Pearson’s *r*** | ***P*** | | **Pearson’s *r*** | ***P*** |
| Frontal pole | -0.34 | 0.21 | | -0.10 | 0.72 |
| Superior frontal gyrus | -0.40 | 0.14 | | -0.29 | 0.29 |
| Rostral middle frontal gyrus | -0.30 | 0.28 | | -0.25 | 0.37 |
| Caudal middle frontal gyrus | -0.16 | 0.58 | | -0.11 | 0.70 |
| Pars orbitalis | -0.13 | 0.63 | | -0.17 | 0.55 |
| Pars opercularis | -0.30 | 0.27 | | -0.22 | 0.42 |
| Pars triangularis | -0.49 | 0.06 | | -0.43 | 0.11 |
| Lateral orbitofrontal cortex | -0.19 | 0.50 | | -0.10 | 0.73 |
| Medial orbitofrontal cortex | -0.44 | 0.10 | | -0.30 | 0.28 |
| Temporal pole | -0.40 | 0.14 | | -0.43 | 0.11 |
| Entorhinal cortex | -0.19 | 0.50 | | -0.23 | 0.40 |
| Parahippocampal cortex | -0.27 | 0.34 | | -0.25 | 0.37 |
| Hippocampus | 0.14 | 0.61 | | 0.15 | 0.60 |
| Amygdala | -0.04 | 0.89 | | -0.11 | 0.70 |
| Inferior temporal gyrus | -0.39 | 0.15 | | -0.35 | 0.20 |
| Fusiform gyrus | -0.56 | 0.03* | | -0.54 | 0.04* |
| Middle temporal gyrus | -0.13 | 0.65 | | -0.11 | 0.70 |
| Banks of the superior temporal sulcus | -0.22 | 0.42 | | -0.09 | 0.74 |
| Superior temporal gyrus | -0.31 | 0.26 | | -0.3 | 0.27 |
| Transverse temporal gyrus | -0.06 | 0.84 | | -0.15 | 0.59 |
| Supramarginal gyrus | -0.26 | 0.35 | | -0.21 | 0.45 |
| Insular cortex | -0.33 | 0.23 | | -0.41 | 0.13 |
| Rostral anterior cingulate cortex | -0.34 | 0.22 | | -0.32 | 0.24 |
| Caudal anterior cingulate cortex | -0.37 | 0.17 | | -0.24 | 0.38 |
| Posterior cingulate cortex | -0.30 | 0.29 | | -0.19 | 0.49 |
| Isthmus of the cingulum | 0.14 | 0.62 | | 0.15 | 0.59 |
| Precuneus | -0.15 | 0.60 | | -0.09 | 0.75 |
| Paracentral gyrus | -0.56 | 0.03* | | -0.53 | 0.04* |
| Postcentral gyrus | -0.50 | 0.06 | | -0.33 | 0.23 |
| Precentral gyrus | -0.57 | 0.03* | | -0.54 | 0.04* |
| Superior parietal lobule | -0.46 | 0.08 | | -0.30 | 0.27 |
| Inferior parietal lobule | -0.19 | 0.50 | | -0.18 | 0.51 |
| Lateral occipital cortex | -0.3 | 0.27 | | -0.16 | 0.58 |
| Cuneus | -0.36 | 0.18 | | -0.30 | 0.29 |
| Pericalcarine cortex | -0.38 | 0.16 | | -0.28 | 0.32 |
| Lingual gyrus | -0.39 | 0.15 | | -0.32 | 0.25 |
| Thalamus | -0.03 | 0.92 | | -0.03 | 0.93 |
| Caudate | 0.02 | 0.95 | | 0.05 | 0.85 |
| Putamen | -0.11 | 0.69 | | -0.04 | 0.88 |
| Pallidum | 0.27 | 0.32 | | 0.29 | 0.30 |
| Nucleus accumbens | -0.24 | 0.40 | | -0.05 | 0.86 |
| Ventral diencephalon | -0.16 | 0.56 | | -0.21 | 0.44 |
| **eTable 6 Continued.** | | | | | |
|  | ***DVR*** | | **PVC *– DVR*** | | |
| **Right Hemisphere Region** | **Pearson’s *r*** | ***P*** | | **Pearson’s *r*** | ***P*** |
| Frontal pole | -0.13 | 0.65 | | -0.19 | 0.51 |
| Superior frontal gyrus | -0.54 | 0.04* | | -0.20 | 0.48 |
| Rostral middle frontal gyrus | -0.39 | 0.15 | | -0.19 | 0.49 |
| Caudal middle frontal gyrus | -0.44 | 0.10 | | 0.13 | 0.63 |
| Pars orbitalis | -0.64 | 0.01* | | -0.03 | 0.91 |
| Pars opercularis | -0.55 | 0.03* | | -0.03 | 0.91 |
| Pars triangularis | -0.39 | 0.15 | | -0.07 | 0.81 |
| Lateral orbitofrontal cortex | -0.29 | 0.29 | | -0.10 | 0.71 |
| Medial orbitofrontal cortex | -0.52 | 0.05 | | -0.29 | 0.30 |
| Temporal pole | -0.48 | 0.07 | | -0.64 | 0.01* |
| Entorhinal cortex | 0.24 | 0.39 | | -0.37 | 0.18 |
| Parahippocampal cortex | -0.22 | 0.43 | | -0.39 | 0.15 |
| Hippocampus | 0.22 | 0.43 | | -0.19 | 0.50 |
| Amygdala | -0.11 | 0.69 | | -0.53 | 0.04 |
| Inferior temporal gyrus | -0.42 | 0.12 | | -0.12 | 0.67 |
| Fusiform gyrus | -0.34 | 0.21 | | -0.29 | 0.30 |
| Middle temporal gyrus | -0.22 | 0.43 | | 0.06 | 0.84 |
| Banks of the superior temporal sulcus | -0.26 | 0.34 | | 0.18 | 0.51 |
| Superior temporal gyrus | -0.43 | 0.11 | | -0.21 | 0.45 |
| Transverse temporal gyrus | -0.15 | 0.59 | | -0.17 | 0.54 |
| Supramarginal gyrus | -0.42 | 0.12 | | 0.17 | 0.54 |
| Insular cortex | -0.32 | 0.24 | | -0.43 | 0.11 |
| Rostral anterior cingulate cortex | -0.27 | 0.34 | | -0.15 | 0.60 |
| Caudal anterior cingulate cortex | -0.33 | 0.23 | | -0.16 | 0.57 |
| Posterior cingulate cortex | -0.32 | 0.24 | | 0.06 | 0.84 |
| Isthmus of the cingulum | 0.25 | 0.37 | | 0.05 | 0.86 |
| Precuneus | -0.20 | 0.48 | | 0.17 | 0.56 |
| Paracentral gyrus | -0.57 | 0.03* | | -0.13 | 0.63 |
| Postcentral gyrus | -0.67 | 0.01* | | -0.05 | 0.85 |
| Precentral gyrus | -0.68 | 0.005* | | -0.03 | 0.92 |
| Superior parietal lobule | -0.36 | 0.18 | | 0.19 | 0.49 |
| Inferior parietal lobule | -0.09 | 0.75 | | 0.19 | 0.50 |
| Lateral occipital cortex | -0.17 | 0.55 | | 0.13 | 0.65 |
| Cuneus | -0.21 | 0.46 | | 0.30 | 0.27 |
| Pericalcarine cortex | -0.27 | 0.33 | | 0.07 | 0.80 |
| Lingual gyrus | -0.27 | 0.33 | | -0.06 | 0.84 |
| Thalamus | 0.06 | 0.83 | | -0.44 | 0.10 |
| Caudate | -0.05 | 0.86 | | -0.24 | 0.40 |
| Putamen | -0.25 | 0.36 | | -0.05 | 0.86 |
| Pallidum | 0.17 | 0.54 | | -0.24 | 0.39 |
| Nucleus accumbens | -0.13 | 0.64 | | -0.62 | 0.01* |
| Ventral diencephalon | -0.18 | 0.53 | | -0.60 | 0.02* |

Pearson’s r and an associated *P*-value (uncorrected for multiple comparisons) are listed. **P* < 0.05 Abbreviations: CSF, cerebrospinal fluid, *DVR*, distribution volume ratio of [^11^C]UCB-J calculated with a cerebellum reference region; AD, Alzheimer’s disease; PET, positron emission tomography; PVC, partial volume correction

| **eTable 7. Correlations between SV2A PET (*DVR*) and CSF SNAP_Total_ in participants with AD** | | | | | |
| --- | --- | --- | --- | --- | --- |
|  | ***DVR*** | | **PVC *– DVR*** | | |
| **Left Hemisphere Region** | **Pearson’s *r*** | ***P*** | | **Pearson’s *r*** | ***P*** |
| Frontal pole | -0.61 | 0.02* | | -0.52 | 0.05 |
| Superior frontal gyrus | -0.58 | 0.02* | | -0.53 | 0.04* |
| Rostral middle frontal gyrus | -0.44 | 0.10 | | -0.44 | 0.10 |
| Caudal middle frontal gyrus | -0.32 | 0.24 | | -0.33 | 0.24 |
| Pars orbitalis | -0.20 | 0.47 | | -0.20 | 0.46 |
| Pars opercularis | -0.32 | 0.24 | | -0.25 | 0.37 |
| Pars triangularis | -0.44 | 0.10 | | -0.55 | 0.03* |
| Lateral orbitofrontal cortex | -0.49 | 0.06 | | -0.34 | 0.22 |
| Medial orbitofrontal cortex | -0.52 | 0.05 | | -0.35 | 0.20 |
| Temporal pole | -0.60 | 0.02* | | -0.57 | 0.03* |
| Entorhinal cortex | -0.58 | 0.02* | | -0.61 | 0.02* |
| Parahippocampal cortex | -0.54 | 0.04* | | -0.52 | 0.05 |
| Hippocampus | -0.25 | 0.36 | | -0.20 | 0.47 |
| Amygdala | -0.39 | 0.16 | | -0.43 | 0.11 |
| Inferior temporal gyrus | -0.37 | 0.18 | | -0.33 | 0.23 |
| Fusiform gyrus | -0.47 | 0.08 | | -0.42 | 0.12 |
| Middle temporal gyrus | -0.32 | 0.24 | | -0.22 | 0.43 |
| Banks of the superior temporal sulcus | -0.29 | 0.30 | | -0.13 | 0.65 |
| Superior temporal gyrus | -0.34 | 0.21 | | -0.30 | 0.27 |
| Transverse temporal gyrus | 0.07 | 0.80 | | 0.10 | 0.73 |
| Supramarginal gyrus | -0.17 | 0.56 | | -0.18 | 0.51 |
| Insular cortex | -0.44 | 0.10 | | -0.46 | 0.09 |
| Rostral anterior cingulate cortex | -0.61 | 0.01* | | -0.58 | 0.02* |
| Caudal anterior cingulate cortex | -0.63 | 0.01* | | -0.45 | 0.10 |
| Posterior cingulate cortex | -0.32 | 0.25 | | -0.34 | 0.21 |
| Isthmus of the cingulum | -0.30 | 0.27 | | -0.32 | 0.24 |
| Precuneus | -0.17 | 0.53 | | -0.17 | 0.54 |
| Paracentral gyrus | -0.48 | 0.07 | | -0.36 | 0.19 |
| Postcentral gyrus | -0.53 | 0.04* | | -0.54 | 0.04* |
| Precentral gyrus | -0.49 | 0.06 | | -0.44 | 0.10 |
| Superior parietal lobule | -0.35 | 0.20 | | -0.35 | 0.20 |
| Inferior parietal lobule | -0.26 | 0.36 | | -0.26 | 0.35 |
| Lateral occipital cortex | -0.17 | 0.54 | | -0.01 | 0.98 |
| Cuneus | -0.40 | 0.14 | | -0.30 | 0.27 |
| Pericalcarine cortex | -0.21 | 0.45 | | -0.15 | 0.59 |
| Lingual gyrus | -0.53 | 0.04* | | -0.45 | 0.09 |
| Thalamus | -0.35 | 0.20 | | -0.43 | 0.11 |
| Caudate | -0.39 | 0.15 | | -0.41 | 0.13 |
| Putamen | -0.38 | 0.16 | | -0.36 | 0.18 |
| Pallidum | -0.50 | 0.06 | | -0.45 | 0.10 |
| Nucleus accumbens | -0.61 | 0.02* | | -0.46 | 0.08 |
| Ventral diencephalon | -0.51 | 0.05 | | -0.48 | 0.07 |
| **eTable 7 Continued.** | | | | | |
|  | ***DVR*** | | **PVC *– DVR*** | | |
| **Right Hemisphere Region** | **Pearson’s *r*** | ***P*** | | **Pearson’s *r*** | ***P*** |
| Frontal pole | -0.48 | 0.07 | | -0.22 | 0.43 |
| Superior frontal gyrus | -0.67 | 0.01* | | -0.18 | 0.51 |
| Rostral middle frontal gyrus | -0.51 | 0.05 | | -0.14 | 0.62 |
| Caudal middle frontal gyrus | -0.44 | 0.10 | | -0.13 | 0.65 |
| Pars orbitalis | -0.51 | 0.05 | | -0.09 | 0.75 |
| Pars opercularis | -0.33 | 0.22 | | -0.07 | 0.80 |
| Pars triangularis | -0.50 | 0.06 | | -0.12 | 0.67 |
| Lateral orbitofrontal cortex | -0.54 | 0.04* | | -0.22 | 0.43 |
| Medial orbitofrontal cortex | -0.59 | 0.02* | | -0.08 | 0.77 |
| Temporal pole | -0.55 | 0.03* | | -0.15 | 0.58 |
| Entorhinal cortex | -0.27 | 0.34 | | 0.06 | 0.84 |
| Parahippocampal cortex | -0.44 | 0.10 | | -0.51 | 0.05 |
| Hippocampus | -0.16 | 0.57 | | -0.16 | 0.56 |
| Amygdala | -0.54 | 0.04* | | -0.06 | 0.84 |
| Inferior temporal gyrus | -0.18 | 0.51 | | -0.21 | 0.45 |
| Fusiform gyrus | -0.41 | 0.13 | | -0.22 | 0.44 |
| Middle temporal gyrus | 0.00 | 1.00 | | -0.19 | 0.50 |
| Banks of the superior temporal sulcus | -0.01 | 0.98 | | -0.01 | 0.98 |
| Superior temporal gyrus | -0.23 | 0.42 | | -0.24 | 0.38 |
| Transverse temporal gyrus | 0.02 | 0.94 | | -0.17 | 0.55 |
| Supramarginal gyrus | -0.16 | 0.57 | | -0.12 | 0.68 |
| Insular cortex | -0.47 | 0.08 | | -0.20 | 0.46 |
| Rostral anterior cingulate cortex | -0.40 | 0.14 | | -0.11 | 0.70 |
| Caudal anterior cingulate cortex | -0.62 | 0.01* | | -0.22 | 0.44 |
| Posterior cingulate cortex | -0.26 | 0.35 | | -0.23 | 0.41 |
| Isthmus of the cingulum | -0.26 | 0.34 | | -0.48 | 0.07 |
| Precuneus | -0.16 | 0.56 | | -0.21 | 0.46 |
| Paracentral gyrus | -0.49 | 0.06 | | -0.26 | 0.35 |
| Postcentral gyrus | -0.47 | 0.07 | | -0.07 | 0.82 |
| Precentral gyrus | -0.57 | 0.03* | | -0.11 | 0.69 |
| Superior parietal lobule | -0.24 | 0.39 | | -0.08 | 0.78 |
| Inferior parietal lobule | 0.04 | 0.89 | | -0.12 | 0.67 |
| Lateral occipital cortex | -0.14 | 0.61 | | -0.18 | 0.53 |
| Cuneus | -0.26 | 0.34 | | -0.30 | 0.27 |
| Pericalcarine cortex | -0.39 | 0.15 | | -0.17 | 0.54 |
| Lingual gyrus | -0.47 | 0.07 | | -0.40 | 0.13 |
| Thalamus | -0.28 | 0.31 | | -0.27 | 0.32 |
| Caudate | -0.35 | 0.20 | | -0.47 | 0.08 |
| Putamen | -0.40 | 0.14 | | -0.24 | 0.39 |
| Pallidum | -0.33 | 0.23 | | -0.32 | 0.25 |
| Nucleus accumbens | -0.41 | 0.13 | | -0.50 | 0.06 |
| Ventral diencephalon | -0.43 | 0.11 | | -0.11 | 0.70 |

Pearson’s r and an associated *P*-value (uncorrected for multiple comparisons) are listed. **P* < 0.05 Abbreviations: CSF, cerebrospinal fluid, *DVR*, distribution volume ratio of [^11^C]UCB-J calculated with a cerebellum reference region; AD, Alzheimer’s disease; PET, Positron Emission Tomography; PVC, partial volume correction; SNAP25, Synaptosomal-Associated Protein-25kDa

| **eTable 8. Correlations between SV2A PET (*DVR*) and CSF SNAP_Long_ in participants with AD** | | | | | |
| --- | --- | --- | --- | --- | --- |
|  | ***DVR*** | | **PVC *– DVR*** | | |
| **Left Hemisphere Region** | **Pearson’s *r*** | ***P*** | | **Pearson’s *r*** | ***P*** |
| Frontal pole | -0.27 | 0.33 | | -0.14 | 0.62 |
| Superior frontal gyrus | -0.37 | 0.18 | | -0.31 | 0.26 |
| Rostral middle frontal gyrus | -0.33 | 0.23 | | -0.52 | 0.05 |
| Caudal middle frontal gyrus | -0.12 | 0.68 | | -0.13 | 0.65 |
| Pars orbitalis | -0.10 | 0.72 | | -0.23 | 0.41 |
| Pars opercularis | -0.15 | 0.59 | | -0.11 | 0.70 |
| Pars triangularis | -0.34 | 0.21 | | -0.41 | 0.13 |
| Lateral orbitofrontal cortex | -0.13 | 0.66 | | -0.03 | 0.91 |
| Medial orbitofrontal cortex | -0.29 | 0.29 | | -0.16 | 0.57 |
| Temporal pole | -0.23 | 0.40 | | -0.26 | 0.34 |
| Entorhinal cortex | -0.08 | 0.77 | | -0.10 | 0.74 |
| Parahippocampal cortex | -0.23 | 0.41 | | -0.19 | 0.51 |
| Hippocampus | 0.17 | 0.55 | | 0.19 | 0.50 |
| Amygdala | 0.10 | 0.73 | | 0.03 | 0.92 |
| Inferior temporal gyrus | -0.29 | 0.29 | | -0.28 | 0.31 |
| Fusiform gyrus | -0.41 | 0.13 | | -0.42 | 0.12 |
| Middle temporal gyrus | -0.12 | 0.68 | | -0.08 | 0.78 |
| Banks of the superior temporal sulcus | -0.09 | 0.74 | | 0.00 | 0.99 |
| Superior temporal gyrus | -0.15 | 0.59 | | -0.17 | 0.56 |
| Transverse temporal gyrus | 0.15 | 0.60 | | 0.13 | 0.66 |
| Supramarginal gyrus | -0.10 | 0.72 | | -0.16 | 0.57 |
| Insular cortex | -0.16 | 0.58 | | -0.23 | 0.42 |
| Rostral anterior cingulate cortex | -0.39 | 0.15 | | -0.39 | 0.15 |
| Caudal anterior cingulate cortex | -0.30 | 0.28 | | -0.21 | 0.45 |
| Posterior cingulate cortex | -0.15 | 0.59 | | -0.16 | 0.57 |
| Isthmus of the cingulum | 0.06 | 0.83 | | 0.04 | 0.89 |
| Precuneus | -0.07 | 0.81 | | -0.02 | 0.94 |
| Paracentral gyrus | -0.43 | 0.11 | | -0.37 | 0.17 |
| Postcentral gyrus | -0.41 | 0.13 | | -0.38 | 0.17 |
| Precentral gyrus | -0.45 | 0.10 | | -0.46 | 0.09 |
| Superior parietal lobule | -0.30 | 0.28 | | -0.26 | 0.35 |
| Inferior parietal lobule | -0.11 | 0.71 | | -0.13 | 0.65 |
| Lateral occipital cortex | -0.13 | 0.64 | | -0.06 | 0.83 |
| Cuneus | -0.23 | 0.40 | | -0.13 | 0.64 |
| Pericalcarine cortex | -0.04 | 0.90 | | 0.02 | 0.93 |
| Lingual gyrus | -0.24 | 0.38 | | -0.20 | 0.48 |
| Thalamus | 0.14 | 0.62 | | 0.07 | 0.80 |
| Caudate | 0.06 | 0.82 | | 0.06 | 0.84 |
| Putamen | 0.04 | 0.88 | | 0.08 | 0.79 |
| Pallidum | 0.02 | 0.95 | | 0.00 | 0.99 |
| Nucleus accumbens | -0.12 | 0.68 | | 0.04 | 0.89 |
| Ventral diencephalon | -0.07 | 0.79 | | -0.09 | 0.76 |
| **eTable 8 Continued.** | | | | | |
|  | ***DVR*** | | **PVC *– DVR*** | | |
| **Right Hemisphere Region** | **Pearson’s *r*** | ***P*** | | **Pearson’s *r*** | ***P*** |
| Frontal pole | -0.09 | 0.75 | | -0.11 | 0.70 |
| Superior frontal gyrus | -0.48 | 0.07 | | -0.19 | 0.50 |
| Rostral middle frontal gyrus | -0.35 | 0.19 | | -0.18 | 0.52 |
| Caudal middle frontal gyrus | -0.30 | 0.27 | | 0.13 | 0.65 |
| Pars orbitalis | -0.50 | 0.06 | | -0.07 | 0.80 |
| Pars opercularis | -0.37 | 0.18 | | -0.04 | 0.87 |
| Pars triangularis | -0.33 | 0.22 | | 0.00 | 0.99 |
| Lateral orbitofrontal cortex | -0.27 | 0.33 | | -0.25 | 0.37 |
| Medial orbitofrontal cortex | -0.41 | 0.13 | | -0.22 | 0.44 |
| Temporal pole | -0.30 | 0.28 | | -0.28 | 0.31 |
| Entorhinal cortex | 0.12 | 0.66 | | -0.04 | 0.89 |
| Parahippocampal cortex | -0.25 | 0.38 | | -0.26 | 0.35 |
| Hippocampus | 0.29 | 0.29 | | 0.03 | 0.92 |
| Amygdala | -0.06 | 0.82 | | -0.15 | 0.60 |
| Inferior temporal gyrus | -0.32 | 0.24 | | -0.03 | 0.91 |
| Fusiform gyrus | -0.32 | 0.24 | | -0.11 | 0.71 |
| Middle temporal gyrus | -0.07 | 0.80 | | 0.05 | 0.85 |
| Banks of the superior temporal sulcus | -0.07 | 0.81 | | 0.32 | 0.24 |
| Superior temporal gyrus | -0.24 | 0.38 | | -0.17 | 0.54 |
| Transverse temporal gyrus | 0.07 | 0.80 | | -0.01 | 0.96 |
| Supramarginal gyrus | -0.22 | 0.42 | | 0.15 | 0.60 |
| Insular cortex | -0.30 | 0.28 | | -0.23 | 0.42 |
| Rostral anterior cingulate cortex | -0.05 | 0.86 | | -0.09 | 0.74 |
| Caudal anterior cingulate cortex | -0.23 | 0.41 | | -0.16 | 0.56 |
| Posterior cingulate cortex | -0.19 | 0.51 | | -0.01 | 0.97 |
| Isthmus of the cingulum | 0.11 | 0.70 | | -0.13 | 0.66 |
| Precuneus | -0.10 | 0.72 | | -0.03 | 0.91 |
| Paracentral gyrus | -0.47 | 0.07 | | -0.13 | 0.65 |
| Postcentral gyrus | -0.54 | 0.04* | | -0.03 | 0.90 |
| Precentral gyrus | -0.54 | 0.04* | | -0.03 | 0.93 |
| Superior parietal lobule | -0.16 | 0.56 | | 0.20 | 0.48 |
| Inferior parietal lobule | 0.05 | 0.85 | | 0.12 | 0.68 |
| Lateral occipital cortex | -0.03 | 0.91 | | 0.09 | 0.74 |
| Cuneus | -0.08 | 0.78 | | 0.14 | 0.61 |
| Pericalcarine cortex | -0.10 | 0.71 | | -0.02 | 0.94 |
| Lingual gyrus | -0.16 | 0.56 | | -0.14 | 0.63 |
| Thalamus | 0.13 | 0.65 | | -0.13 | 0.65 |
| Caudate | 0.03 | 0.92 | | -0.24 | 0.39 |
| Putamen | -0.08 | 0.79 | | -0.14 | 0.61 |
| Pallidum | 0.13 | 0.63 | | -0.09 | 0.74 |
| Nucleus accumbens | -0.04 | 0.88 | | -0.45 | 0.09 |
| Ventral diencephalon | -0.12 | 0.66 | | -0.24 | 0.39 |

Pearson’s r and an associated *P*-value (uncorrected for multiple comparisons) are listed. **P* < 0.05 Abbreviations: CSF, cerebrospinal fluid, *DVR*, distribution volume ratio of [^11^C]UCB-J calculated with a cerebellum reference region; AD, Alzheimer’s disease; PET, Positron Emission Tomography; PVC, partial volume correction; SNAP25, Synaptosomal-Associated Protein-25kDa

| **eTable 9. Correlations between SV2A PET (*DVR*) and CSF neurogranin in participants with AD** | | | | | |
| --- | --- | --- | --- | --- | --- |
|  | ***DVR*** | | **PVC *– DVR*** | | |
| **Left Hemisphere Region** | **Pearson’s *r*** | ***P*** | | **Pearson’s *r*** | ***P*** |
| Frontal pole | 0.05 | 0.84 | | -0.03 | 0.91 |
| Superior frontal gyrus | -0.21 | 0.36 | | -0.25 | 0.27 |
| Rostral middle frontal gyrus | -0.09 | 0.68 | | -0.23 | 0.31 |
| Caudal middle frontal gyrus | -0.05 | 0.83 | | -0.09 | 0.70 |
| Pars orbitalis | -0.18 | 0.43 | | -0.19 | 0.40 |
| Pars opercularis | 0.05 | 0.82 | | -0.04 | 0.86 |
| Pars triangularis | -0.04 | 0.87 | | -0.15 | 0.52 |
| Lateral orbitofrontal cortex | -0.10 | 0.65 | | -0.16 | 0.49 |
| Medial orbitofrontal cortex | -0.09 | 0.71 | | -0.08 | 0.72 |
| Temporal pole | 0.08 | 0.72 | | 0.03 | 0.88 |
| Entorhinal cortex | 0.23 | 0.31 | | 0.21 | 0.36 |
| Parahippocampal cortex | 0.06 | 0.79 | | 0.1 | 0.68 |
| Hippocampus | 0.31 | 0.17 | | 0.27 | 0.24 |
| Amygdala | 0.48 | 0.03* | | 0.46 | 0.03* |
| Inferior temporal gyrus | -0.12 | 0.59 | | -0.1 | 0.68 |
| Fusiform gyrus | -0.28 | 0.22 | | -0.25 | 0.27 |
| Middle temporal gyrus | -0.12 | 0.60 | | -0.12 | 0.62 |
| Banks of the superior temporal sulcus | 0.06 | 0.80 | | 0.09 | 0.71 |
| Superior temporal gyrus | -0.05 | 0.82 | | -0.1 | 0.65 |
| Transverse temporal gyrus | 0.07 | 0.78 | | -0.02 | 0.92 |
| Supramarginal gyrus | -0.03 | 0.90 | | -0.1 | 0.68 |
| Insular cortex | 0.06 | 0.81 | | -0.04 | 0.85 |
| Rostral anterior cingulate cortex | -0.02 | 0.94 | | -0.1 | 0.67 |
| Caudal anterior cingulate cortex | 0.09 | 0.71 | | 0.13 | 0.58 |
| Posterior cingulate cortex | -0.23 | 0.32 | | -0.21 | 0.35 |
| Isthmus of the cingulum | -0.22 | 0.34 | | -0.21 | 0.35 |
| Precuneus | -0.22 | 0.35 | | -0.17 | 0.47 |
| Paracentral gyrus | -0.35 | 0.12 | | -0.38 | 0.09 |
| Postcentral gyrus | -0.16 | 0.48 | | -0.17 | 0.45 |
| Precentral gyrus | -0.20 | 0.40 | | -0.26 | 0.26 |
| Superior parietal lobule | -0.21 | 0.35 | | -0.16 | 0.50 |
| Inferior parietal lobule | -0.15 | 0.51 | | -0.17 | 0.46 |
| Lateral occipital cortex | -0.30 | 0.19 | | -0.28 | 0.21 |
| Cuneus | -0.36 | 0.11 | | -0.35 | 0.12 |
| Pericalcarine cortex | -0.09 | 0.70 | | -0.05 | 0.82 |
| Lingual gyrus | -0.31 | 0.17 | | -0.33 | 0.14 |
| Thalamus | 0.39 | 0.08 | | 0.37 | 0.10 |
| Caudate | 0.10 | 0.65 | | 0.13 | 0.59 |
| Putamen | 0.23 | 0.32 | | 0.25 | 0.28 |
| Pallidum | 0.55 | 0.01* | | 0.54 | 0.01* |
| Nucleus accumbens | 0.00 | 0.98 | | 0.11 | 0.64 |
| Ventral diencephalon | 0.41 | 0.07 | | 0.37 | 0.09 |
| **eTable 9 Continued.** | | | | | |
|  | ***DVR*** | | **PVC *– DVR*** | | |
| **Right Hemisphere Region** | **Pearson’s *r*** | ***P*** | | **Pearson’s *r*** | ***P*** |
| Frontal pole | -0.22 | 0.35 | | -0.17 | 0.47 |
| Superior frontal gyrus | -0.16 | 0.48 | | -0.17 | 0.45 |
| Rostral middle frontal gyrus | 0.00 | 1.00 | | -0.01 | 0.97 |
| Caudal middle frontal gyrus | 0.01 | 0.96 | | -0.01 | 0.95 |
| Pars orbitalis | -0.22 | 0.34 | | -0.24 | 0.30 |
| Pars opercularis | 0.04 | 0.86 | | -0.09 | 0.71 |
| Pars triangularis | 0.03 | 0.89 | | -0.05 | 0.81 |
| Lateral orbitofrontal cortex | -0.05 | 0.82 | | -0.11 | 0.63 |
| Medial orbitofrontal cortex | -0.02 | 0.94 | | -0.06 | 0.80 |
| Temporal pole | 0.06 | 0.81 | | 0.04 | 0.87 |
| Entorhinal cortex | 0.31 | 0.17 | | 0.31 | 0.18 |
| Parahippocampal cortex | -0.02 | 0.93 | | -0.05 | 0.83 |
| Hippocampus | 0.48 | 0.03* | | 0.47 | 0.03* |
| Amygdala | 0.37 | 0.10 | | 0.36 | 0.11 |
| Inferior temporal gyrus | -0.22 | 0.35 | | -0.25 | 0.27 |
| Fusiform gyrus | -0.13 | 0.58 | | -0.10 | 0.67 |
| Middle temporal gyrus | -0.11 | 0.63 | | -0.18 | 0.44 |
| Banks of the superior temporal sulcus | 0.15 | 0.52 | | 0.13 | 0.57 |
| Superior temporal gyrus | -0.01 | 0.97 | | -0.12 | 0.61 |
| Transverse temporal gyrus | 0.30 | 0.18 | | 0.25 | 0.28 |
| Supramarginal gyrus | 0.05 | 0.84 | | 0.00 | 0.98 |
| Insular cortex | 0.07 | 0.78 | | -0.04 | 0.87 |
| Rostral anterior cingulate cortex | 0.08 | 0.72 | | 0.05 | 0.84 |
| Caudal anterior cingulate cortex | 0.03 | 0.89 | | 0.10 | 0.66 |
| Posterior cingulate cortex | -0.21 | 0.37 | | -0.13 | 0.57 |
| Isthmus of the cingulum | -0.06 | 0.80 | | -0.04 | 0.86 |
| Precuneus | -0.22 | 0.34 | | -0.17 | 0.45 |
| Paracentral gyrus | -0.33 | 0.14 | | -0.33 | 0.14 |
| Postcentral gyrus | -0.19 | 0.42 | | -0.26 | 0.26 |
| Precentral gyrus | -0.23 | 0.32 | | -0.29 | 0.20 |
| Superior parietal lobule | -0.09 | 0.71 | | -0.07 | 0.76 |
| Inferior parietal lobule | 0.01 | 0.97 | | 0.00 | 0.99 |
| Lateral occipital cortex | -0.15 | 0.51 | | -0.16 | 0.50 |
| Cuneus | -0.20 | 0.37 | | -0.14 | 0.54 |
| Pericalcarine cortex | -0.09 | 0.70 | | -0.07 | 0.76 |
| Lingual gyrus | -0.15 | 0.51 | | -0.16 | 0.49 |
| Thalamus | 0.34 | 0.13 | | 0.33 | 0.15 |
| Caudate | 0.03 | 0.90 | | 0.03 | 0.89 |
| Putamen | 0.11 | 0.62 | | 0.12 | 0.62 |
| Pallidum | 0.47 | 0.03* | | 0.48 | 0.03* |
| Nucleus accumbens | 0.20 | 0.39 | | 0.30 | 0.18 |
| Ventral diencephalon | 0.36 | 0.11 | | 0.34 | 0.14 |

Pearson’s r and an associated *P*-value (uncorrected for multiple comparisons) are listed. **P* < 0.05 Abbreviations: CSF, cerebrospinal fluid, *DVR*, distribution volume ratio of [^11^C]UCB-J calculated with a cerebellum reference region; AD, Alzheimer’s disease; PVC, partial volume correction

| **eTable 10. Correlations between SV2A PET (*DVR*) and CSF NFL in participants with AD** | | | | | |
| --- | --- | --- | --- | --- | --- |
|  | ***DVR*** | | **PVC *– DVR*** | | |
| **Left Hemisphere Region** | **Pearson’s *r*** | ***P*** | | **Pearson’s *r*** | ***P*** |
| Frontal pole | -0.16 | 0.49 | | 0.10 | 0.67 |
| Superior frontal gyrus | -0.30 | 0.18 | | -0.16 | 0.48 |
| Rostral middle frontal gyrus | -0.19 | 0.41 | | 0.07 | 0.75 |
| Caudal middle frontal gyrus | -0.30 | 0.18 | | -0.21 | 0.35 |
| Pars orbitalis | -0.14 | 0.56 | | 0.02 | 0.95 |
| Pars opercularis | -0.15 | 0.50 | | -0.06 | 0.79 |
| Pars triangularis | -0.18 | 0.44 | | 0.02 | 0.92 |
| Lateral orbitofrontal cortex | -0.01 | 0.96 | | 0.12 | 0.62 |
| Medial orbitofrontal cortex | -0.13 | 0.58 | | -0.02 | 0.94 |
| Temporal pole | -0.15 | 0.51 | | -0.03 | 0.89 |
| Entorhinal cortex | -0.17 | 0.45 | | -0.12 | 0.62 |
| Parahippocampal cortex | -0.41 | 0.06 | | -0.38 | 0.09 |
| Hippocampus | -0.18 | 0.43 | | -0.21 | 0.37 |
| Amygdala | 0.08 | 0.71 | | 0.11 | 0.65 |
| Inferior temporal gyrus | -0.27 | 0.23 | | -0.16 | 0.49 |
| Fusiform gyrus | -0.39 | 0.08 | | -0.29 | 0.21 |
| Middle temporal gyrus | -0.17 | 0.45 | | -0.09 | 0.70 |
| Banks of the superior temporal sulcus | -0.30 | 0.19 | | -0.21 | 0.37 |
| Superior temporal gyrus | -0.26 | 0.26 | | -0.16 | 0.48 |
| Transverse temporal gyrus | -0.17 | 0.47 | | -0.16 | 0.50 |
| Supramarginal gyrus | -0.25 | 0.27 | | -0.13 | 0.59 |
| Insular cortex | -0.31 | 0.17 | | -0.25 | 0.27 |
| Rostral anterior cingulate cortex | -0.17 | 0.46 | | -0.09 | 0.69 |
| Caudal anterior cingulate cortex | -0.28 | 0.21 | | -0.09 | 0.69 |
| Posterior cingulate cortex | -0.29 | 0.20 | | -0.18 | 0.44 |
| Isthmus of the cingulum | 0.03 | 0.90 | | 0.07 | 0.76 |
| Precuneus | -0.13 | 0.57 | | -0.03 | 0.88 |
| Paracentral gyrus | -0.31 | 0.18 | | -0.23 | 0.32 |
| Postcentral gyrus | -0.38 | 0.09 | | -0.18 | 0.42 |
| Precentral gyrus | -0.41 | 0.06 | | -0.28 | 0.22 |
| Superior parietal lobule | -0.25 | 0.28 | | -0.12 | 0.62 |
| Inferior parietal lobule | -0.11 | 0.64 | | -0.03 | 0.91 |
| Lateral occipital cortex | -0.28 | 0.22 | | -0.12 | 0.60 |
| Cuneus | -0.34 | 0.13 | | -0.25 | 0.28 |
| Pericalcarine cortex | -0.34 | 0.13 | | -0.20 | 0.38 |
| Lingual gyrus | -0.36 | 0.11 | | -0.27 | 0.23 |
| Thalamus | -0.31 | 0.17 | | -0.32 | 0.16 |
| Caudate | -0.31 | 0.17 | | -0.29 | 0.20 |
| Putamen | -0.28 | 0.23 | | -0.20 | 0.38 |
| Pallidum | 0.05 | 0.83 | | 0.03 | 0.89 |
| Nucleus accumbens | -0.18 | 0.44 | | -0.05 | 0.84 |
| Ventral diencephalon | -0.16 | 0.48 | | -0.18 | 0.44 |
| **eTable 10 Continued.** | | | | | |
|  | ***DVR*** | | **PVC *– DVR*** | | |
| **Right Hemisphere Region** | **Pearson’s *r*** | ***P*** | | **Pearson’s *r*** | ***P*** |
| Frontal pole | -0.17 | 0.46 | | -0.03 | 0.89 |
| Superior frontal gyrus | -0.44 | 0.04* | | -0.28 | 0.22 |
| Rostral middle frontal gyrus | -0.34 | 0.13 | | -0.12 | 0.61 |
| Caudal middle frontal gyrus | -0.44 | 0.05 | | -0.33 | 0.14 |
| Pars orbitalis | -0.26 | 0.26 | | -0.13 | 0.59 |
| Pars opercularis | -0.32 | 0.15 | | -0.19 | 0.42 |
| Pars triangularis | -0.45 | 0.04* | | -0.30 | 0.18 |
| Lateral orbitofrontal cortex | -0.07 | 0.75 | | 0.08 | 0.73 |
| Medial orbitofrontal cortex | 0.03 | 0.89 | | 0.12 | 0.61 |
| Temporal pole | -0.23 | 0.32 | | -0.18 | 0.43 |
| Entorhinal cortex | -0.03 | 0.89 | | 0.06 | 0.78 |
| Parahippocampal cortex | -0.54 | 0.01* | | -0.49 | 0.02* |
| Hippocampus | -0.08 | 0.75 | | -0.06 | 0.79 |
| Amygdala | -0.01 | 0.96 | | 0.03 | 0.91 |
| Inferior temporal gyrus | -0.38 | 0.09 | | -0.28 | 0.22 |
| Fusiform gyrus | -0.38 | 0.09 | | -0.30 | 0.18 |
| Middle temporal gyrus | -0.35 | 0.12 | | -0.26 | 0.25 |
| Banks of the superior temporal sulcus | -0.41 | 0.06 | | -0.35 | 0.12 |
| Superior temporal gyrus | -0.44 | 0.05 | | -0.37 | 0.10 |
| Transverse temporal gyrus | -0.49 | 0.02* | | -0.43 | 0.05 |
| Supramarginal gyrus | -0.58 | 0.01* | | -0.46 | 0.04* |
| Insular cortex | -0.30 | 0.18 | | -0.27 | 0.23 |
| Rostral anterior cingulate cortex | -0.23 | 0.31 | | -0.17 | 0.47 |
| Caudal anterior cingulate cortex | -0.17 | 0.47 | | -0.01 | 0.97 |
| Posterior cingulate cortex | -0.20 | 0.38 | | -0.11 | 0.63 |
| Isthmus of the cingulum | -0.18 | 0.43 | | -0.15 | 0.53 |
| Precuneus | -0.20 | 0.39 | | -0.11 | 0.63 |
| Paracentral gyrus | -0.39 | 0.08 | | -0.28 | 0.21 |
| Postcentral gyrus | -0.55 | 0.01* | | -0.42 | 0.06 |
| Precentral gyrus | -0.52 | 0.02* | | -0.41 | 0.07 |
| Superior parietal lobule | -0.43 | 0.05 | | -0.31 | 0.18 |
| Inferior parietal lobule | -0.35 | 0.11 | | -0.30 | 0.19 |
| Lateral occipital cortex | -0.35 | 0.12 | | -0.27 | 0.23 |
| Cuneus | -0.45 | 0.04* | | -0.39 | 0.08 |
| Pericalcarine cortex | -0.40 | 0.07 | | -0.28 | 0.22 |
| Lingual gyrus | -0.43 | 0.05 | | -0.34 | 0.13 |
| Thalamus | -0.23 | 0.33 | | -0.22 | 0.34 |
| Caudate | -0.37 | 0.10 | | -0.36 | 0.11 |
| Putamen | -0.40 | 0.08 | | -0.39 | 0.08 |
| Pallidum | -0.05 | 0.83 | | -0.04 | 0.87 |
| Nucleus accumbens | -0.24 | 0.29 | | -0.15 | 0.50 |
| Ventral diencephalon | -0.14 | 0.55 | | -0.15 | 0.51 |

Pearson’s r and an associated *P*-value (uncorrected for multiple comparisons) are listed. **P* < 0.05 Abbreviations: CSF, cerebrospinal fluid, *DVR*, distribution volume ratio of [^11^C]UCB-J calculated with a cerebellum reference region; AD, Alzheimer’s disease; PET, positron emission tomography; PVC, partial volume correction; NFL, Neurofilament Light Chain

| **eTable 11. Correlations between SV2A PET (*DVR*) and CSF GAP-43 in participants with AD** | | | | | |
| --- | --- | --- | --- | --- | --- |
|  | ***DVR*** | | **PVC *– DVR*** | | |
| **Left Hemisphere Region** | **Pearson’s *r*** | ***P*** | | **Pearson’s *r*** | ***P*** |
| Frontal pole | -0.15 | 0.51 | | -0.09 | 0.69 |
| Superior frontal gyrus | -0.40 | 0.07 | | -0.39 | 0.08 |
| Rostral middle frontal gyrus | -0.26 | 0.26 | | -0.29 | 0.20 |
| Caudal middle frontal gyrus | -0.17 | 0.47 | | -0.22 | 0.33 |
| Pars orbitalis | -0.29 | 0.20 | | -0.29 | 0.21 |
| Pars opercularis | -0.09 | 0.69 | | -0.16 | 0.50 |
| Pars triangularis | -0.24 | 0.30 | | -0.27 | 0.24 |
| Lateral orbitofrontal cortex | -0.32 | 0.16 | | -0.32 | 0.16 |
| Medial orbitofrontal cortex | -0.32 | 0.15 | | -0.30 | 0.18 |
| Temporal pole | -0.18 | 0.43 | | -0.23 | 0.32 |
| Entorhinal cortex | -0.04 | 0.85 | | -0.06 | 0.79 |
| Parahippocampal cortex | -0.19 | 0.41 | | -0.15 | 0.53 |
| Hippocampus | 0.07 | 0.75 | | 0.05 | 0.83 |
| Amygdala | 0.17 | 0.47 | | 0.13 | 0.57 |
| Inferior temporal gyrus | -0.28 | 0.22 | | -0.26 | 0.26 |
| Fusiform gyrus | -0.44 | 0.05 | | -0.43 | 0.05 |
| Middle temporal gyrus | -0.25 | 0.27 | | -0.23 | 0.32 |
| Banks of the superior temporal sulcus | -0.06 | 0.79 | | 0.01 | 0.96 |
| Superior temporal gyrus | -0.25 | 0.27 | | -0.28 | 0.21 |
| Transverse temporal gyrus | -0.05 | 0.84 | | -0.10 | 0.67 |
| Supramarginal gyrus | -0.11 | 0.63 | | -0.16 | 0.49 |
| Insular cortex | -0.26 | 0.25 | | -0.33 | 0.14 |
| Rostral anterior cingulate cortex | -0.35 | 0.12 | | -0.40 | 0.07 |
| Caudal anterior cingulate cortex | -0.29 | 0.21 | | -0.20 | 0.39 |
| Posterior cingulate cortex | -0.32 | 0.15 | | -0.33 | 0.14 |
| Isthmus of the cingulum | -0.26 | 0.25 | | -0.28 | 0.22 |
| Precuneus | -0.21 | 0.36 | | -0.19 | 0.40 |
| Paracentral gyrus | -0.48 | 0.03* | | -0.49 | 0.02* |
| Postcentral gyrus | -0.36 | 0.11 | | -0.34 | 0.14 |
| Precentral gyrus | -0.38 | 0.09 | | -0.43 | 0.05 |
| Superior parietal lobule | -0.23 | 0.31 | | -0.16 | 0.49 |
| Inferior parietal lobule | -0.20 | 0.39 | | -0.19 | 0.40 |
| Lateral occipital cortex | -0.33 | 0.14 | | -0.27 | 0.23 |
| Cuneus | -0.48 | 0.03* | | -0.46 | 0.03* |
| Pericalcarine cortex | -0.29 | 0.20 | | -0.25 | 0.28 |
| Lingual gyrus | -0.51 | 0.02* | | -0.52 | 0.02* |
| Thalamus | 0.19 | 0.42 | | 0.14 | 0.56 |
| Caudate | -0.13 | 0.56 | | -0.13 | 0.57 |
| Putamen | -0.02 | 0.92 | | -0.01 | 0.96 |
| Pallidum | 0.32 | 0.16 | | 0.32 | 0.15 |
| Nucleus accumbens | -0.20 | 0.37 | | -0.13 | 0.58 |
| Ventral diencephalon | 0.12 | 0.60 | | 0.10 | 0.67 |
| **eTable 11 Continued.** | | | | | |
|  | ***DVR*** | | **PVC *– DVR*** | | |
| **Right Hemisphere Region** | **Pearson’s *r*** | ***P*** | | **Pearson’s *r*** | ***P*** |
| Frontal pole | -0.34 | 0.13 | | -0.19 | 0.42 |
| Superior frontal gyrus | -0.43 | 0.05 | | -0.41 | 0.06 |
| Rostral middle frontal gyrus | -0.27 | 0.24 | | -0.22 | 0.34 |
| Caudal middle frontal gyrus | -0.16 | 0.48 | | -0.18 | 0.43 |
| Pars orbitalis | -0.45 | 0.04* | | -0.41 | 0.06 |
| Pars opercularis | -0.15 | 0.51 | | -0.23 | 0.32 |
| Pars triangularis | -0.17 | 0.45 | | -0.21 | 0.37 |
| Lateral orbitofrontal cortex | -0.40 | 0.07 | | -0.39 | 0.08 |
| Medial orbitofrontal cortex | -0.30 | 0.19 | | -0.31 | 0.17 |
| Temporal pole | -0.15 | 0.51 | | -0.17 | 0.46 |
| Entorhinal cortex | 0.20 | 0.38 | | 0.21 | 0.36 |
| Parahippocampal cortex | -0.20 | 0.38 | | -0.22 | 0.34 |
| Hippocampus | 0.32 | 0.16 | | 0.32 | 0.15 |
| Amygdala | 0.15 | 0.51 | | 0.14 | 0.55 |
| Inferior temporal gyrus | -0.32 | 0.15 | | -0.36 | 0.11 |
| Fusiform gyrus | -0.28 | 0.23 | | -0.27 | 0.23 |
| Middle temporal gyrus | -0.21 | 0.37 | | -0.28 | 0.22 |
| Banks of the superior temporal sulcus | 0.01 | 0.95 | | -0.04 | 0.87 |
| Superior temporal gyrus | -0.24 | 0.29 | | -0.36 | 0.11 |
| Transverse temporal gyrus | 0.10 | 0.67 | | 0.09 | 0.69 |
| Supramarginal gyrus | -0.09 | 0.69 | | -0.18 | 0.44 |
| Insular cortex | -0.22 | 0.33 | | -0.31 | 0.17 |
| Rostral anterior cingulate cortex | -0.20 | 0.39 | | -0.22 | 0.35 |
| Caudal anterior cingulate cortex | -0.24 | 0.29 | | -0.14 | 0.53 |
| Posterior cingulate cortex | -0.29 | 0.21 | | -0.25 | 0.28 |
| Isthmus of the cingulum | -0.16 | 0.48 | | -0.17 | 0.46 |
| Precuneus | -0.24 | 0.29 | | -0.24 | 0.29 |
| Paracentral gyrus | -0.49 | 0.02* | | -0.48 | 0.03* |
| Postcentral gyrus | -0.41 | 0.07 | | -0.44 | 0.04* |
| Precentral gyrus | -0.44 | 0.05 | | -0.50 | 0.02* |
| Superior parietal lobule | -0.17 | 0.47 | | -0.14 | 0.55 |
| Inferior parietal lobule | -0.04 | 0.85 | | -0.09 | 0.71 |
| Lateral occipital cortex | -0.17 | 0.47 | | -0.19 | 0.41 |
| Cuneus | -0.29 | 0.20 | | -0.23 | 0.32 |
| Pericalcarine cortex | -0.29 | 0.20 | | -0.23 | 0.31 |
| Lingual gyrus | -0.36 | 0.11 | | -0.35 | 0.12 |
| Thalamus | 0.15 | 0.52 | | 0.11 | 0.65 |
| Caudate | -0.17 | 0.47 | | -0.18 | 0.44 |
| Putamen | -0.17 | 0.45 | | -0.18 | 0.44 |
| Pallidum | 0.22 | 0.33 | | 0.26 | 0.26 |
| Nucleus accumbens | 0.01 | 0.95 | | 0.06 | 0.80 |
| Ventral diencephalon | 0.09 | 0.70 | | 0.07 | 0.76 |

Pearson’s r and an associated *P*-value (uncorrected for multiple comparisons) are listed. **P* < 0.05 Abbreviations: CSF, cerebrospinal fluid, *DVR*, distribution volume ratio of [^11^C]UCB-J calculated with a cerebellum reference region; AD, Alzheimer’s disease; PET, Positron Emission Tomography; PVC, partial volume correction; GAP-43, Growth Associated Protein 43

**3. eFigures**

C

U

A

β

-

AD

A

β

+

0

1000

2000

3000

4000

CSF SV2A pg/mL

*P* < 0.0001

**eFigure 1. CSF SV2A levels in amyloid negative cognitively unimpaired (n = 20) and Alzheimer’s disease (n = 20) in discovery cohort 1.** *P* value is for an unpaired t-test. Dots represent the CSF protein concentration for each participant. Box plots display the median, upper and lower quartile, and upper and lower extreme. Abbreviations: AD, Alzheimer’s disease; CSF, cerebrospinal fluid; CU, cognitively unimpaired; SV2A, synaptic vesicle glycoprotein 2A

C

U

A

β

-

C

U

A

β

+

AD

A

β

+

0

1000

2000

3000

4000

CSF SV2A ng/mL

*P* < 0.0001

*P* < 0.0001

**eFigure 2. CSF SV2A levels in amyloid negative cognitively unimpaired (n = 32), amyloid positive cognitively unimpaired (n = 14) and Alzheimer’s disease (n = 31) in discovery cohort 2.** *P* values are for post hoc unpaired t-tests. Dots represent the CSF protein concentration for each participant. Box plots display the median, upper and lower quartile, and upper and lower extreme. Abbreviations: AD, Alzheimer’s disease; CSF, cerebrospinal fluid; CU, cognitively unimpaired; SV2A, synaptic vesicle glycoprotein 2A


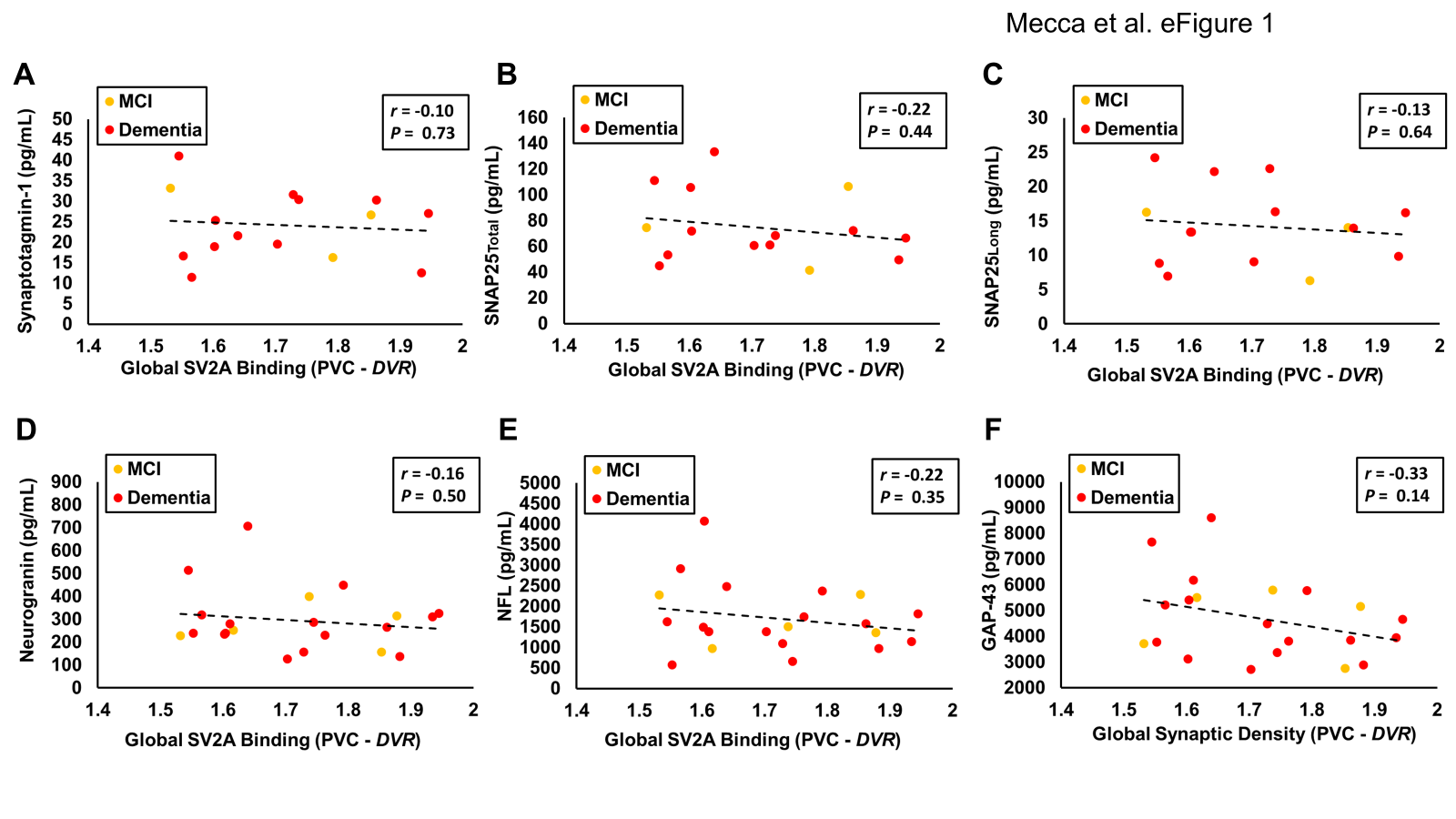


**eFigure 3. Correlation of partial volume corrected SV2A binding (PVC-*DVR*) and CSF measures of synaptic or neuronal damage in participants with AD.** Global SV2A binding calculated after partial volume correction of [^11^C]UCB-J PET images (PVC-*DVR*) was plotted with **(A)** Synaptotagmin-1 (n = 15), **(B)** SNAP25_Total_ (n = 15), **(C)** SNAP25_Long_ (n = 15), **(D)** Neurogranin (n = 21), **(E)** NFL (n = 21), and **(F)** GAP-43 (n = 21) concentration in the group of participants with AD. Disease stage of MCI is represented with yellow dots and dementia is represented with red dots. Data points for individual participants and a regression line (dotted) are plotted. *r* and *P*-values are displayed for Pearson’s correlations. Abbreviations: AD, Alzheimer’s disease, *DVR*, distribution volume ratio of [^11^C]UCB-J calculated with a whole cerebellum reference region; MCI, mild cognitive impairment; SV2A, synaptic vesicle glycoprotein 2A; CSF, cerebrospinal fluid; CN, cognitively normal; SNAP25, Synaptosomal-Associated Protein-25kDa; NFL, Neurofilament Light Chain; GAP-43, Growth Associated Protein 43


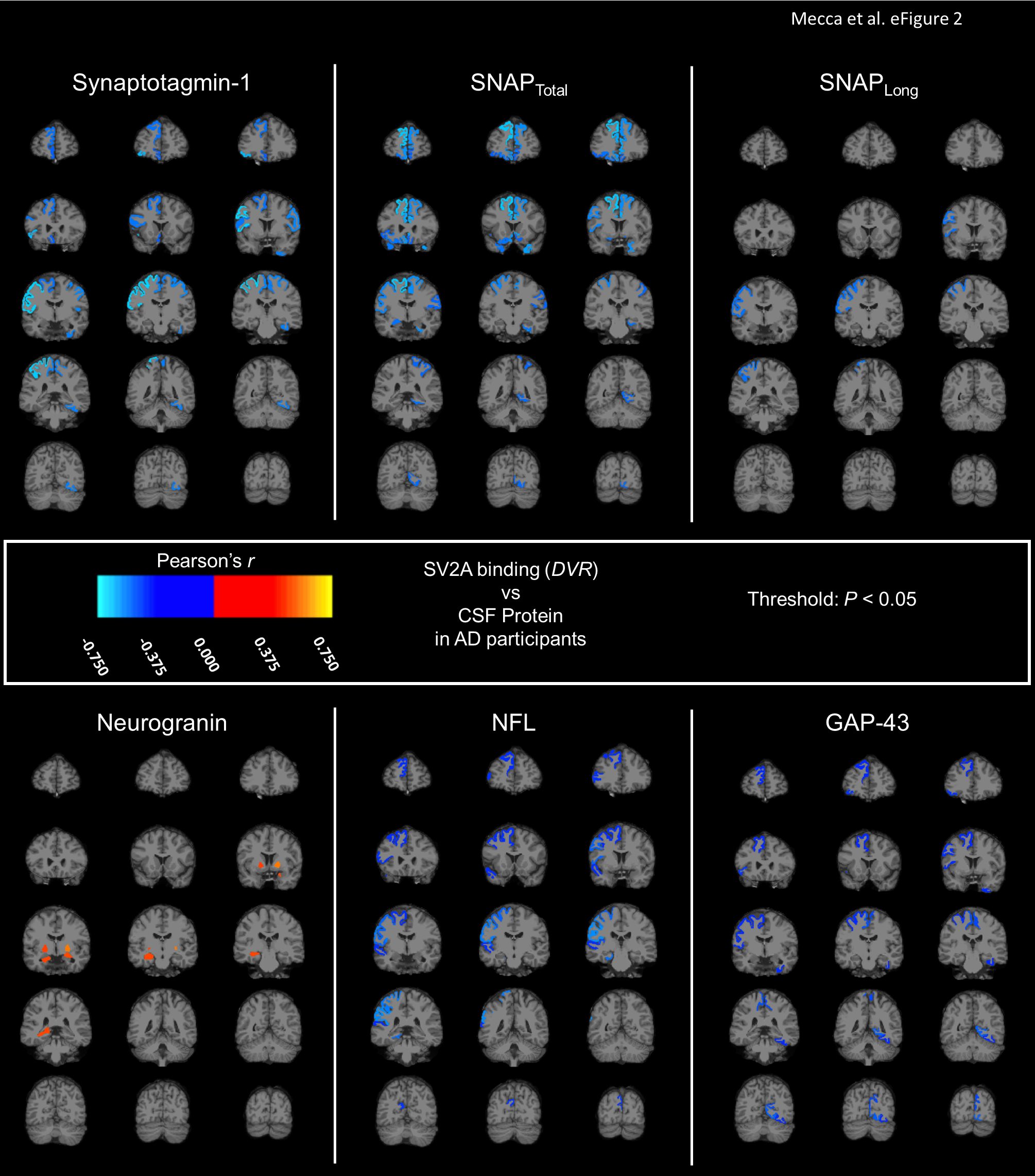


**eFigure 4.** **Regional correlations of SV2A binding (*DVR)* and CSF measures of synaptic or neuronal damage in participants with AD.** In the group of AD participants, Pearson’s *r* was calculated for the correlations between SV2A binding in each brain region and the concentration of each CSF protein biomarker including: **(A)** Synaptotagmin-1 (n = 15), **(B)** SNAP25_Total_ (n = 15), **(C)** SNAP25_Long_ (n = 15), **(D)** Neurogranin (n = 21), **(E)** NFL (n = 21), and **(F)** GAP-43 (n = 21). Brain maps were created by producing images with the voxels in each brain region set uniformly to the calculated Pearson’s *r* for that region and overlaid on an MNI template T1 MRI. The color scale represents Pearson’s *r*, which is displayed only for regions that had an uncorrected *P* < 0.05. Abbreviations: AD, Alzheimer’s disease; CSF, cerebrospinal fluid; *DVR*, distribution volume ratio of [^11^C]UCB-J calculated with a whole cerebellum reference region; SNAP25, Synaptosomal-Associated Protein-25kDa; NFL, Neurofilament Light Chain; GAP-43, Growth Associated Protein 43


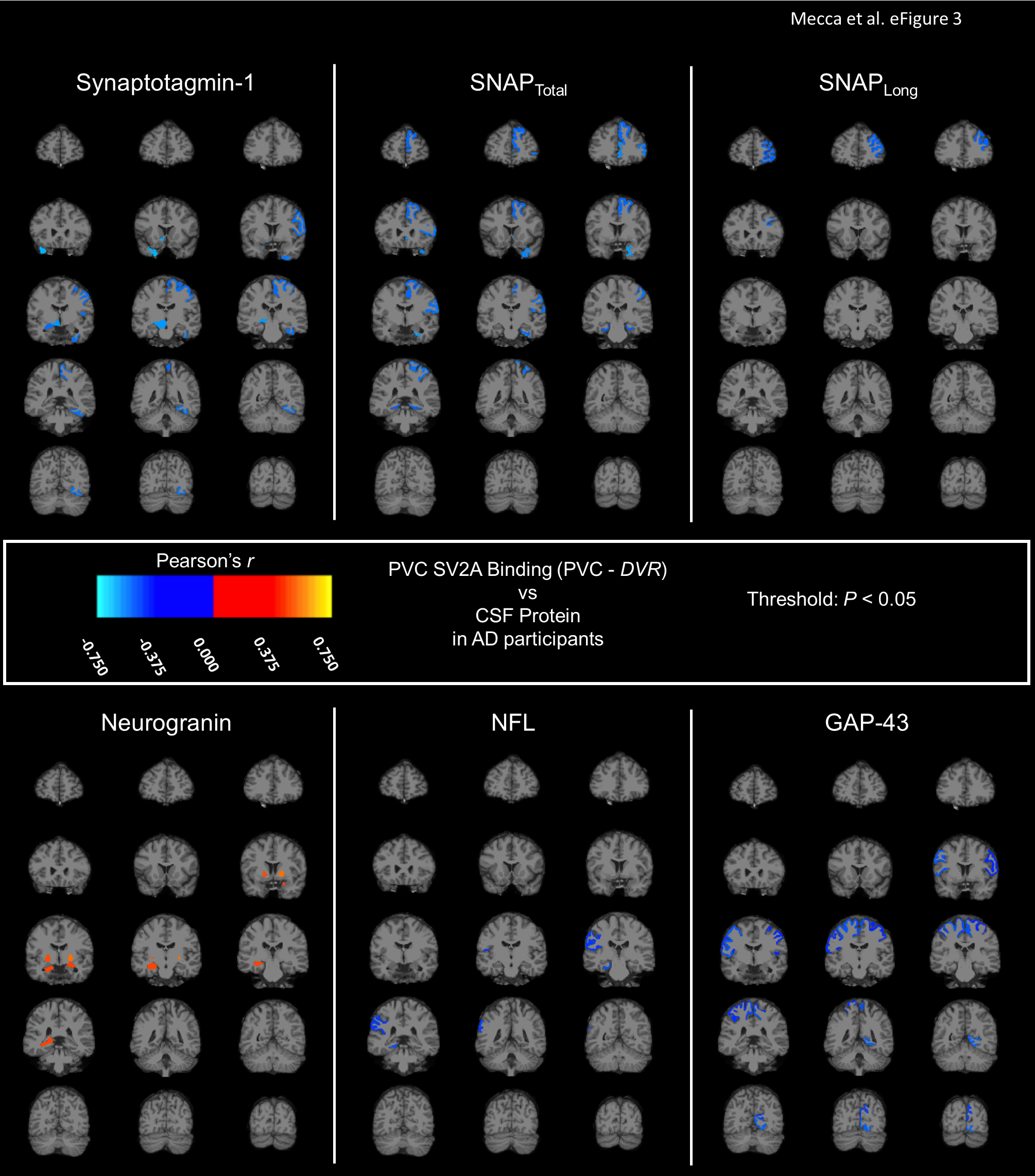


**eFigure 5. Regional correlations of SV2A binding (PVC-*DVR*) and CSF measures of synaptic or neuronal damage in participants with AD.** After partial volume correction of [^11^C]UCB-J PET images, Pearson’s *r* was calculated for the correlations between SV2A binding (PVC-*DVR*) in each brain region and the concentration of each CSF protein biomarker including: **(A)** Synaptotagmin-1 (n = 15), **(B)** SNAP25_Total_ (n = 15), **(C)** SNAP25_Long_ (n = 15), **(D)** Neurogranin (n = 21), **(E)** NFL (n = 21), and **(F)** GAP-43 (n = 21) in the group of participants with AD. Brain maps were created by producing images with the voxels in each brain region set uniformly to the calculated Pearson’s *r* for that region and overlaid on an MNI template T1 MRI. The color scale represents Pearson’s *r*, which is displayed only for regions that had an uncorrected *P* < 0.05. Abbreviations: AD, Alzheimer’s disease; CSF, cerebrospinal fluid; *DVR*, distribution volume ratio of [^11^C]UCB-J calculated with a whole cerebellum reference region; PVC, partial volume correction; SNAP25, Synaptosomal-Associated Protein-25kDa; NFL, Neurofilament Light Chain; GAP-43, Growth Associated Protein 43

**4. References**

[1] Reiman E, Chen K, Liu X, Bandy D, Yu M, Lee W, et al. Fibrillar amyloid-{beta} burden in cognitively normal people at 3 levels of genetic risk for Alzheimer's disease. Proc Natl Acad Sci U S A. 2009;106:6820-5.

[2] Mecca AP, Barcelos NM, Wang S, Bruck A, Nabulsi N, Planeta-Wilson B, et al. Cortical beta-amyloid burden, gray matter, and memory in adults at varying APOE epsilon4 risk for Alzheimer's disease. Neurobiol Aging. 2017;61:207-14.

[3] Dubois B, Feldman HH, Jacova C, Hampel H, Molinuevo JL, Blennow K, et al. Advancing research diagnostic criteria for Alzheimer's disease: the IWG-2 criteria. Lancet Neurol. 2014;13:614-29.

[4] Erlandsson K, Buvat I, Pretorius PH, Thomas BA, Hutton BF. A review of partial volume correction techniques for emission tomography and their applications in neurology, cardiology and oncology. Phys Med Biol. 2012;57:R119-59.

[5] Shidahara M, Thomas BA, Okamura N, Ibaraki M, Matsubara K, Oyama S, et al. A comparison of five partial volume correction methods for Tau and Amyloid PET imaging with [18F]THK5351 and [11C]PIB. Ann Nucl Med. 2017;31:563-9.

[6] Mecca AP, O'Dell RS, Sharp ES, Banks ER, Bartlett HH, Zhao W, et al. Synaptic density and cognitive performance in Alzheimer's disease: A PET imaging study with [(11) C]UCB-J. Alzheimers Dement. 2022.

[7] de Jong HW, van Velden FH, Kloet RW, Buijs FL, Boellaard R, Lammertsma AA. Performance evaluation of the ECAT HRRT: an LSO-LYSO double layer high resolution, high sensitivity scanner. Phys Med Biol. 2007;52:1505-26.

[8] Carson RE, Barker W, Liow J-S, Adler S, Johnson C. Design of a motion-compensation OSEM List-mode Algorithm for Resolution-Recovery Reconstruction of the HRRT. IEEE Nucl Sci Symp Conf Rec. 2003;M16-6.

[9] Jin X, Mulnix T, Gallezot JD, Carson RE. Evaluation of motion correction methods in human brain PET imaging--a simulation study based on human motion data. Medical physics. 2013;40:102503.

[10] Finnema SJ, Nabulsi NB, Mercier J, Lin SF, Chen MK, Matuskey D, et al. Kinetic evaluation and test-retest reproducibility of [(11)C]UCB-J, a novel radioligand for positron emission tomography imaging of synaptic vesicle glycoprotein 2A in humans. J Cereb Blood Flow Metab. 2018;38:2041-52.

[11] Fischl B. FreeSurfer. Neuroimage. 2012;62:774-81.

[12] Mecca AP, Chen MK, O'Dell RS, Naganawa M, Toyonaga T, Godek TA, et al. In vivo measurement of widespread synaptic loss in Alzheimer's disease with SV2A PET. Alzheimers Dement. 2020;16:974-82.

[13] Innis RB, Cunningham VJ, Delforge J, Fujita M, Gjedde A, Gunn RN, et al. Consensus nomenclature for in vivo imaging of reversibly binding radioligands. J Cereb Blood Flow Metab. 2007;27:1533-9.

[14] Wu Y, Carson RE. Noise reduction in the simplified reference tissue model for neuroreceptor functional imaging. J Cereb Blood Flow Metab. 2002;22:1440-52.

[15] Mertens N, Maguire RP, Serdons K, Lacroix B, Mercier J, Sciberras D, et al. Validation of Parametric Methods for [(11)C]UCB-J PET Imaging Using Subcortical White Matter as Reference Tissue. Mol Imaging Biol. 2020;22:444-52.

[16] Rossano S, Toyonaga T, Finnema SJ, Naganawa M, Lu Y, Nabulsi N, et al. Assessment of a white matter reference region for (11)C-UCB-J PET quantification. J Cereb Blood Flow Metab. 2019:271678X19879230.

[17] Matuskey D, Tinaz S, Wilcox KC, Naganawa M, Toyonaga T, Dias M, et al. Synaptic Changes in Parkinson Disease Assessed with in vivo Imaging. Ann Neurol. 2020;87:329-38.
